# Modeling Obesity in Norway (The MOON Study): A Decision-Analytic Approach—Prevalence, Costs, and Years of Life Lost

**DOI:** 10.1177/0272989X20971589

**Published:** 2020-11-30

**Authors:** Gudrun M. W. Bjørnelv, Vidar Halsteinli, Bård E. Kulseng, Diana Sonntag, Rønnaug A. Ødegaard

**Affiliations:** Regional Centre for Health Care Development, St. Olavs Hospital, Trondheim, Norway; Department of Public Health and Nursing, NTNU, Trondheim, Norway; Regional Centre for Health Care Development, St. Olavs Hospital, Trondheim, Norway; Department of Public Health and Nursing, NTNU, Trondheim, Norway; Regional Center for Obesity Research and Innovation, Department of Surgery, St. Olavs Hospital, Trondheim, Norway; Department of Clinical Molecular Medicine, NTNU, Trondheim, Norway; Mannheim Institute of Public Health, Social and Preventive Medicine, Mannheim Medical Faculty of the Heidelberg University, Mannheim, Germany; Department of Health Sciences, University of York, UK; Regional Center for Obesity Research and Innovation, Department of Surgery, St. Olavs Hospital, Trondheim, Norway; Department of Clinical Molecular Medicine, NTNU, Trondheim, Norway

**Keywords:** cost and cost analysis, economic evaluation, Markov model, obesity

## Abstract

**Background:**

Limited knowledge exists on the expected long-term effects and cost-effectiveness of initiatives aiming to reduce the burden of obesity.

**Aim:**

To develop a Norwegian obesity-focused disease-simulation model: the MOON model.

**Material and Methods:**

We developed a Markov model and simulated a Norwegian birth cohort’s movement between the health states “normal weight,”“overweight,”“obese 1,”“obese 2,” and “dead” using a lifetime perspective. Model input was estimated using longitudinal data from health surveys and real-world data (RWD) from local and national registers (*N* = 99,348). The model is deterministic and probabilistic and stratified by gender. Model validity was assessed by estimating the cohort’s expected prevalence, health care costs, and mortality related to overweight and obesity.

**Results:**

Throughout the cohort’s life, the prevalence of overweight increased steadily and stabilized at 45% at 45 y of age. The number of obese 1 and 2 individuals peaked at age 75 y, when 44% of women and 35% of men were obese. The incremental costs per person associated with obesity was highest in older ages and, when accumulated over the lifetime, higher among women (€12,118, €9,495–€15,047) than men (€6,646, €5,252–€10,900). On average, obesity shortened the life expectancy of women/men in the whole cohort by 1.31/1.08 y. The life expectancy for normal-weight women/men at age 30 was 83.31/80.31. The life expectancy was reduced by 1.05/0.65 y if the individual was overweight, obese (2.87/2.71 y), or obese 2 (4.06/4.83 y).

**Conclusion:**

The high expected prevalence of obesity in the future will lead to substantial health care costs and large losses in life-years. This underscores the need to implement interventions to reduce the burden of obesity; the MOON model will enable economic evaluations for a wide range of interventions.

The prevalence of overweight and obesity in children and adults worldwide has increased rapidly during the past 4 decades.^1^ Obesity is linked to an increased incidence of a number of chronic diseases (e.g., type 2 diabetes, cardiovascular diseases, osteoarthritis, respiratory disease, and certain cancers), which leads to increased morbidity and a marked loss in years of life.^2–6^

Globally, the awareness of obesity and its consequences is increasing, and efforts to reduce the burden of obesity through prevention and treatment are therefore rising on the political agenda.^7^ Economic arguments are increasingly used across several countries to inform decision making and to support efficient resource allocation.^8^

Economic evaluations within obesity call for a long time perspective and comparison of multiple alternatives for the following reasons.^9–12^ First, the negative outcomes related to obesity tend to be later in life but are also apparent for childhood obesity, in which the increased risk of diseases is primarily driven by the persistence of obesity from childhood to adulthood.^13–16^ Second, prevention and treatment of obesity can be provided through a wide range of policy initiatives and complex multimodal interventions that—because of time and resource constraints—cannot be compared within a single clinical trial. Analytical frameworks such as disease simulation models (DSMs) can enable the evaluation of a range of interventions, considering both effects and costs while taking a long time perspective.^17–21^

Several obesity-focused DSMs have been previously created.^17,18^ One of the earliest examples of model-based economic evaluations is the Assessing Cost-Effectiveness in Obesity (ACE-Obesity) program in Australia (2006),^22^ in which a broad range of preventive and treatment interventions were evaluated to guide resource allocation using pathway analyses. More recently, as part of the Childhood Obesity Intervention Cost-Effectiveness Study (CHOICES) in the United States, Ward et al.^16^ used longitudinal data and developed a model in which they simulated individual-level height and weight trajectories for participants aged 2 to 35 y while accounting for secular trends. In Germany, Sonntag et al.^23,24^ used a more traditional modeling approach whereby they used cross-sectional data to developed a Markov model using the body mass index (BMI) categories “normal weight,”“overweight,” and “obese” as health states to estimate the expected lifetime costs of childhood obesity.

Because of the differences between countries regarding the epidemiology of diseases, health care systems, and cost levels, DSMs cannot be directly applied across countries.^25,26^ Building on the German approach, we have developed the first Markov model within a Norwegian context using longitudinal health survey data linked to registry data, which provides a unique opportunity to make valid, data-heavy DSMs. In the present study, Modeling Obesity in Norway (MOON), an obesity-focused DSM, was built to enable future economic evaluations in a Norwegian context.

In the present article, we first describe the details of how we developed the MOON model, with a focus on model transparency. We showcase the model and test its validity by first estimating the expected prevalence and incremental health care costs of overweight and obesity throughout the lifetime for a cohort of Norwegian 2-y-olds and, second, by estimating the cohorts’ expected years of life loss (YLL) due to obesity through scenario analyses.

## Materials and Methods

### Model Structure

**Figure 1:**
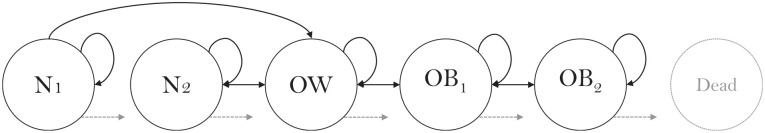
Schematic of the Markov model. The light gray arrows indicate how individuals can move to “dead” from each of the other health states.

We developed a Markov model that included the following health states: normal weight (NW_1_ and NW_2_), overweight (OW), obese grade 1 (OB_1_), obese grade 2 (OB_2_), and dead (circles in Figure 1). NW_1_ represents “always NW,” whereas NW_2_ represents “NW but previously OW or OB.” In the current article, we assume that the health states N1 and N2 have the same transition probabilities, health care costs, and mortality rates. However, in future analyses, we have the flexibility to separate those who have always been normal weight from those who are normal weight but who have previously been overweight and/or obese.

Health states were defined based on the World Health Organization cutoffs, which are based on the individuals’ BMI (kg/m^2^).^27^ In adults, a BMI of 18 to 25, 25 to 30, 30 to 35, or 35+ kg/m^2^ is classified as NW, OW, OB_1_, or OB_2_, respectively. Because these cutoffs are insufficient at classifying children, we used the International Obesity Task Force for children (2–17 y), which classifies children into the same health states, but in which the cutoffs are age and gender specific; for example, a 2-y-old girl is classified as NW, OW, OB_1_, or OB_2_ if she has a BMI of 14.96 to 18.09, 18.09 to 19.81, 19.81 to 21.13, or 21.13+kg/m^2^, respectively.^28^

A cohort of children entered the model at 2 y of age and were distributed between the health states according to a representative sample of 2-y-olds in Norway in 2002. Accordingly, 89.83%, 8.98%, 0.86%, and 0.33% were NW, OW, OB_1_, or OB_2_. The model simulates 1-y cycles, over 98 y (until individuals have died or reach 100 y old). For each cycle, the cohort can move between the health states along transition possibilities (arrows in Figure 1); for example, if a person is OW, she or he can either stay OW, become OB_1_, NW_2_, or die. We track the number of individuals in each health state, per year, and estimate the cohorts’ expected annual health care costs and number of deaths. This was done by multiplying the number of persons in each health state by the health care cost and mortality rate of that health state. All input parameters in the model (transition probabilities, mortality rates, and health care costs) varied with age, which coincided with the number of the cycle. We simulated a cohort of 55,120 individuals—the size of the Norwegian birth cohort born in 2018—in which 48% were female and 52% were male.^29^ The model was built in Excel (Office 2016).

### Model Input

#### Transition probabilities

Transition probabilities were estimated using 4 patient-level longitudinal data sets: 1) Trondheim municipality (T-MU), 2) the Child Growth Study (CGS), 3) Young-HUNT, and 4) Adult HUNT.

T-MU contains all children born in the Trondheim municipality between 1999 and 2017. In Norway, all children are followed up at health stations at predefined time points (12 times during the first 2 y and at approximately 2, 4, 6, 8, and 13 y of age). We extracted data from the children’s electronic medical records, from which data were available from 2003 to 2017. The CGS contains a representative sample of Norwegian children who were aged 8 y in 2010 and information from their electronic medical records from birth. The Nord-Trøndelag Health Study (the HUNT Study) contains several surveys targeting the population in the former county Nord Trøndelag. It is representative of the Norwegian population with regard to geography, economy, industry, sources of income, age distribution, morbidity, and mortality.^30^ We used information from Young-HUNT 1, 2, and 3 and adult HUNT 1, 2, and 3. In the Young-HUNT 1 survey (1995–1997), 10,000 adolescents in secondary school (aged 13–15 y) and high school (aged 15–19 y) were invited to participate. A 90% participation rate was achieved. The Young-HUNT 2 survey (1999–2000) was a follow-up study of Young-HUNT 1, in which all adolescents in the last 2 y of high school (aged 17–19 y) were invited. A participation rate of 77% was obtained. In Young-HUNT 3 (2006–2008), 10,000 adolecents in secondary and high school were invited, and an 87% response rate was obtained.^31^ Young-HUNT 1, 2, and 3 can be linked to adult HUNT 3. Adult HUNT 1, 2, and 3 were performed in 3 waves: in 1984–1986, 1995–1997, and 2006–2008. In the adult HUNT surveys, all citizens living in Nord-Trøndelag County aged >20 y were invited. The participation rates in the surveys were 88%, 71%, and 54%, respectively.^32^ We constructed 2 data sets based on the HUNT surveys: an adolescent data set containing individuals who participated in either Young-HUNT 1, 2, or 3 and could be linked either through the Young-HUNT surveys or to adult HUNT 3 and an adult data set, containing individuals wo participated in adult HUNT 1, 2, and/or 3. From T-MU, CGS, and HUNT, we received data on the individuals’ age, gender, height, and weight. Data on height was measured without shoes, whereas weight was measured with light indoor clothing.

We created 3 longitudinal data sets (children, adolescents, and adults) by restricting all data sets to contain only individuals who had 2 or more observations including height and weight. Individual observations did not need to be complete (e.g., appear both in Young-HUNT 1, 2, 3 and HUNT 3 to be part of the adolescent data) but could appear in Young-HUNT 1 and then in HUNT 3, or in Young-HUNT 1 and then only in Young-HUNT 2. Eighteen individuals participated in both Young-HUNT 3 and HUNT 3. These were included in the adolescent data set. Similarly, to be part of the adult data set, individuals could appear in HUNT 1, 2, or 3 if they appeared in a minimum of 2 waves. Altogether, we had 2 or more observations on 99,348 individuals (average of 2.84 observations); 35,506 individuals from T-MU and 2909 individuals from CGS (a total of 38,415 individuals in the children data set), 3097 individuals in the adolescent HUNT data set, and 57,836 individuals in the adult HUNT data set. See Supplementary Appendix 1 for a schematic of the data and more information regarding participation in the different data sets.

To exploit the longitudinal structure of the data, transition probabilities were estimated using parametric survival analyses. We conducted separate analyses for 3 different age groups: children (aged 2–13 y), adolescents (aged 13–30 y), and adults (aged 20–102 y), using data from the T-MU and CGS (for children); Young-HUNT 1, 2, and 3 and adult HUNT 3 (for adolescents); and adult HUNT 1, 2, and 3 (for adults). In the MOON model, we used data from the childhood analyses between the ages 2 and 12 y, adolescent analyses between the ages of 13 and 19 y, and adulthood analyses for ages 20+y. We chose to use transition probabilities in the MOON from the starting age of the survival analyses. The age range is therefore higher in the survival analyses than what it was in the MOON model (e.g., for adolescents, we use data from the survival analyses starting at the age of 13 but end at 19 y since the survival data for the adults start at the age of 20 y).

**Figure 2:**
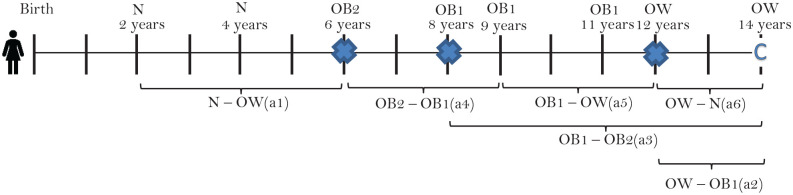
Schematic of a hypothetical person, built on observations in the data set, and his or her contribution in the different survival analyses (a 1–6). N = NW, OW = OW, OB1 = OB_1_ OB2 = OB_2_, 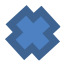 = event, 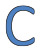 = censoring.

Because data were measured between large time gaps (∼10 y in the HUNT studies, ∼4 y between Young HUNT 1 and 2, and ∼11 y between Young HUNT 1 and adult HUNT 3), we interpolated the data by estimating the height and weight between measurement points. For the children cohort (T-MU and COIS), data were gathered at sufficiently close time gaps, and no interpolation was performed. In the adolescent populations and the adult populations, we interpolated values at a yearly basis between the measurements (i.e., ∼4 y between Young HUNT 1 and Young HUNT 2, ∼7 y between Young HUNT 2 and adult HUNT 3, and ∼10 y between HUNT 1, HUNT 2, and HUNT 3). We did not interpolate between Young-Hunt 3 and HUNT 3, because the average time gap for the 18 individuals who participated in both surveys was 1 y. We assumed a linear increase in height until the age of 18 y, when height was assumed to be constant, and a linear increase in weight between all measurements (both adolescents and adults; see supplementary Appendix 2). For all 3 age groups, we conducted 6 separate survival analyses. Three analyses estimated the probability of progressing (moving to higher BMI classes) 1) from N1/N2→OW, 2) from OW→OB_1_, and 3) from OB_1_→OB_2_), and 3 analyses estimated the probability of regressing (moving to lower BMI classes) 4) from OB_2_→OB_1_, 5) from OB_1_→OW, and 6) OW→N2 (see Figure 1). We restructured the data to enable survival analyses by defining a start time, whether an individual had an event or was censored, and a time-to-event or time-to-censoring. The start time was set when an individual had his or her first observation as either NW (analysis 1), OW (analyses 2 and 6), OB_1_ (analyses 3 and 5), or OB2 (analysis 4): 1 person could thus inform several of the survival analyses (see Figure 2).

**Figure 3:**
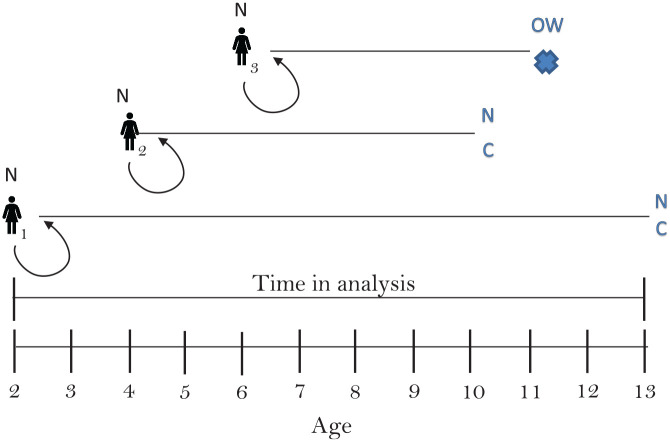
Schematic of 3 hypothetical persons in survival analysis 1 (from NW to OW). Person 1 enters the model as NW at the age 2 y and stays NW until the age 13 y, when she is censored (lost to follow-up). Person 2 enters the model as NW at the age of 4 y and stays NW until the age of 10, when she is censored. Person 3 enters the model as NW at the age of 6 y and stays NW until the age of 11, when she becomes OW (has an event), 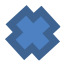 = event, 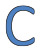 = censoring.

An individual had an event when she or he either moved up a weight class (e.g., from NW to OW) or down a weight class (e.g., from OW to NW), depending on which of the 6 analyses we conducted. Individuals were censored when they no longer had an observation. We used age as the time axis in the analysis and allowed for delayed entry, meaning that individuals entered the survival model when the model reached their age. See Figure 3 for an example.

For the 18 survival analyses (6 for each age group), we assessed the goodness-of-fit from 6 distributions (the exponential, Weibull, log-normal, log-logistic, Gompertz, or gamma distribution)^33^ using the Akaike information criterion and the Bayesian information criterion.^34,35^ We conducted analyses for both the whole cohort and when stratifying by gender; model selection was performed for the whole cohort, and the same specifications of the survival models were assumed for women and men.

#### Mortality

We estimated age-, gender-, and BMI-specific mortality rates by using age- and gender-specific national life tables for the Norwegian population in 2017. We multiplied the mortality rates with age- and BMI-specific hazard ratios to estimate the additional mortality from overweight and obesity. The hazard ratios data were estimated from the Global BMI Mortality collaboration.^36^ See Supplementary Appendix 3.

#### Health care costs

We used 3 data sets to estimate health care costs: HUNT-3, the Norwegian Patient Registry (NPR), and Control and Payment of Health Reimbursement Register (KUHR).^37,38^ From HUNT-3, we had access to information on 50,410 respondents and their height, weight, age, gender, smoking status, and marital status. These were linked to NPR and KUHR. In Norway, health care is partly funded based on activity (activity-based funding), and close to 100% of hospital and general practitioners’ (GP) activity is recorded in NPR and KUHR. From NPR, we had access to information on inpatient and outpatient use of secondary care, including somatic and psychiatric care. We used cost weights from the Norwegian activity-based funding system to estimate hospital costs. From KUHR, we had access to information on use of GPs, and because GPs are partly financed through claims, we used these claims to estimate the cost of GPs. Costs were estimated in 2009 EUROs; 1 EURO = 8.7285 NOK = 0.7197 USD.^39^ For details, see Supplementary Appendix 4.

Data from HUNT-3 were collected between 2006 and 2008, while we used data from NPR and KUHR collected in 2009; we therefore selected people from HUNT-3 who were still alive and living in Norway on 1 January 2010 (*N* = 49,714). Variables on health care costs were nonnegatively skewed continuous numbers and contained a large quantity of nonusers, and we estimated the incremental health care costs between those who were NW (reference group), OW, OB_1_, or OB_2_ using 2-part models with a logistic regression (first part) and a generalized linear model with a log link and a gamma distribution (second part).^40,41^ In the 2-part model, we included covariates that are commonly used in the obesity literature: age (continuous), smoking status (never-smoker, previous smoker, daily smoker, and occasional smoker), and marital status (married or registered partner, never married, widow/widower, or divorced/separated). We conducted stratified models for women and men. We predicted the average marginal effects at representative values per year from the age of 20 to 80 y and used these as inputs in the MOON model.^42^ Costs between the age of 80 and 100 y were assumed to be constant at the level predicted in 80-y-olds. For details, see Supplementary Appendix 4.

All statistical analyses were performed in STATA version 15.

### Model Output

#### Base-case analyses

We estimated the expected prevalence, health care costs, and mortality throughout the lifetime for a cohort of Norwegian 2-y-olds. We assumed that trends in obesity prevalence and mortality during the last decades (1980s–2000s) were applicable to future predictions (i.e., mirroring the data). The prevalence was expressed as the annual expected percentage of the cohort—still alive—who were NW, OW, OB_1_, or OB_2_. Costs were estimated as the annual expected survival-adjusted incremental health care costs of OW, OB_1_, and OB_2_ and as the cumulative expected survival-adjusted incremental health care costs of OW, OB_1_, and OB_2_ during the cohort’s lifetime. Annual health care costs were estimated by first multiplying the number of individuals who were in a health state at a certain time (age) with the per-person cost (from the 2-part models) of being in that health state at that time (age). Incremental costs of being OW, OB_1_, and OB_2_ were estimated by subtracting the expected costs for those being OW, OB_1_, or OB_2_ from the expected costs for those being NW. Cumulative costs were estimated by summarizing the annual expected costs over the cohort’s lifetime. Numbers are primarily displayed as the expected costs per person in the cohort, estimated by dividing the annual or cumulative costs by the start population in the model (*n* = 26,458 for women and *n* = 28,662 for men). The model is built to enable discounting; we display results without discounting and when discounting costs at a 4% level.

#### Sensitivity analyses

The model was built both deterministic and probabilistic. In the deterministic analyses, we used point estimates of the population means as input parameters. In the probabilistic sensitivity analyses (PSA), input parameters were predefined as distributions with predefined variability. The choice of distributions and size of the variability are displayed in Table 1 for transition probabilities (defined from the survival analyses). For mortality rates, we used a log-normal distribution and variability from the Global BMI Mortality collaboration (Supplementary Appendix S1), and for health care costs, we used gamma distributions with variability from the 2-part models (Supplementary Appendix s6–s13).^34^ When performing the PSA, we allowed for the model to draw randomly from the predefined distributions to make a single realization of the model. We repeated the procedure (random draw and realization) using 1000 iterations. Using results from the PSA, we estimated the 95% confidence bounds (CBs) as the 2.5% and 97.5% observation for relevant variables from the 1000 iterations.

**Table 1:** Description of Which Distributions We Used in the Survival Models, Their Corresponding Parameters, and Average Transition Probabilities for the Different Transition Possibilities (Arrows in Figure 1) for Children, Adolescents, and Adults^a^

		Women^b^			Men^b^		
Age Group	Distribution	*n*	Parameter	Transition Probability^c^	n	Parameter	Transition Probability^c^
N - OW							
2–12 y	Log-normal	17,312	(3.16, 1.22)	2.72%	18,508	(3.27, 1.19)	2.32%
13–19 y	Weibull	1495	(−5.75, 1.85)	1.85%	1222	(−6.95, 2.42)	1.80%
20 + y	Log-logistic	19,599	(0.45, 0.04)	3.25%	14,499	(0.50, 0.06)	3.86%
OW – OB_1_							
2–12 y	Log-normal	3562	(3.32, 1.51)	2.85%	3279	(3.28, 1.31)	2.56%
13 – 19 y	Weibull	560	(−5.22, 1.77)	2.67%	483	(−4.53, 1.54)	3.28%
20 + y	Log-logistic	18,062	(0.61, 0.04)	2.38%	18,966	(0.73, 0.03)	1.63%
OB_1_– OB_2_							
2–12 y	Log-normal	689	(3.35, 1.62)	2.95%	610	(3.44, 1.62)	2.73%
13–19 y	Gompertz	164	(0.12, 0.02)	3.40%	139	(0.02, 0.05)	4.98%
20 + y	Gompertz	7469	(−0.02, 0.05)	2.35%	5501	(0.01, 0.02)	1.38%
OW – N							
2–12 y	Log-normal	3562	(1.64, 0.95)	13.17%	3279	(1.44, 0.91)	15.97%
13–19 y	Gompertz	560	(−0.30, 0.22)	7.83%	483	(−0.36, 0.22)	6.88%
20 + y	Gompertz	18,062	(0.04, 0.00^d^)	1.00%	18,966	(0.05, 0.00^e^)	1.10%
OB_1_– OW							
2–12 y	Log-normal	689	(1.28, 0.92)	18.11%	610	(1.26, 0.89)	18.88%
13–19 y	Gompertz	164	(−0.43, 0.31)	8.10%	139	(−0.32, 0.16)	5.59%
20 + y	Gompertz	7469	(0.03, 0.00^f^)	0.96%	5501	(0.05, 0.00^g^)	1.95%
OB_2_– OB_1_							
2–12 y	Log-normal	222	(1.27, 0.77)	20.93%	194	(1.37, 0.84)	18.22%
13–19 y	Gompertz	41	(−0.35, 0.48)	14.43%	23	(−0.16, 0.16)	8.46%
20 + y	Gompertz	2191	(0.02, 0.00^h^)	2.17%	846	(0.04, 0.00^i^)	2.79%

a For yearly transition probabilities, see Supplementary Appendix 5.

b For the probabilistic sensitivity analysis, we sampled using Cholesky decomposition.^31^ Covariance matrixes (necessary to perform probabilistic sensitivity analyses) are available upon request from the author.

c Transition probabilities given as the average yearly values for the relevant age group. For example, the average yearly transition probability between NW and OW was 2.72% for females between the ages of 2 and 12 y, based on the yearly probabilities 0.49% +1.70% +2.46% +2.88% +3.11% +3.24% +3.31% +3.33% +3.33% +3.31%)/10 ≈ 2.72%. In the model, we used yearly transition probabilities, which varied with age.

d Value = 0.00141.

e Value = 0.00086.

f Value = 0.00227.

g Value = 0.00160.

h Value = 0.00782.

i Value = 0.00414.

#### Model validation

We assessed the model’s face validity through a continuous and close collaboration with clinicians, internal validity by testing the models’ mathematical accuracy through control cells and by performing a walkthrough of the model with independent researchers, and external validity by comparing results from the model with the input data (dependent comparison).^43^ For details, see Supplementary Appendix 6.

#### Scenario analyses

We performed 6 scenario analyses. First, we estimated the expected YLL attributable to OB_1_ and OB_2_ in the cohort through 2 scenarios: 1) when eliminating OB_2_ and 2) when eliminating OB_2_ and OB_1_. The scenarios were performed by manipulating transition probabilities from the health state OB_1_ to OB_2_ (scenario 1) or from OW to OB_1_ (scenario 2) to be zero (i.e., no transition to OB_2_ or OB_1_, respectively). In the scenarios, we set OB_2_ individuals as OB_1_ individuals at age 2 (scenario 1) and OB_1_ and OB_2_ individuals as OW at age 2 (scenario 2). The probability of staying in OB_1_ increased by the reduced probability of going from OB_1_ to OB_2_ (scenario 1) and from OW to OB_1_ (scenario 2) so that the sum of transition probabilities from the health states was always 1. We also ran 4 scenario analyses in which we estimated the expected YLL for individuals aged 30 y conditional on their weight category. Analyses were performed by estimating the life expectancy when all individuals at age 30 was NW (scenario 3), OW (scenario 4), OB_1_ (scenario 5), or OB_2_ (scenario 6). YLL was estimated by subtracting the life expectancy for the OW, OB_1_, or OB_2_ from that of the NW. In scenarios 3 to 6, individuals could move between health states according to transition probabilities as used in the MOON model after the age of 30; thus, individuals who were NW at age 30 could progress to OW, OB_1_, or OB_2_ throughout their lifetime, and likewise, individuals who were OB_2_ at age 30 could regress to OB_1_, OW, and NW throughout their lifetime.

## Results

### Transition between Health States

Transition between health states was frequent in young ages but decreased and stabilized at low levels in older ages (Table 1; Supplementary Appendix 5).

### Prevalence

**Figures 4:**
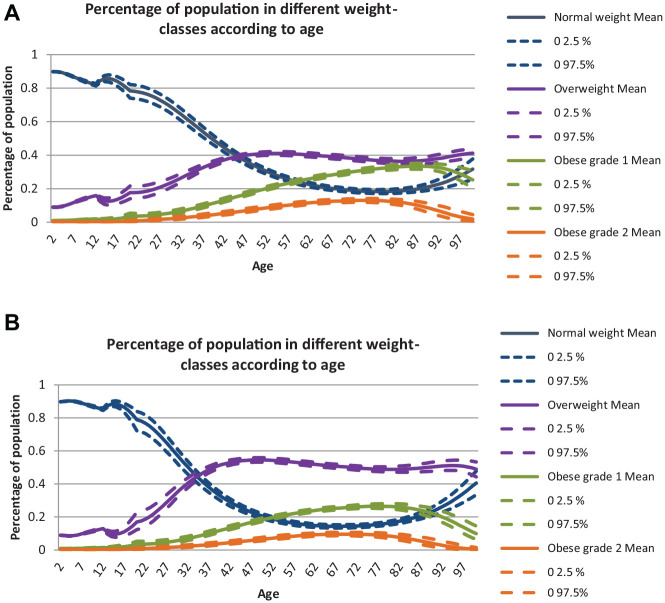
Predicted proportions (and 95% confidence bounds) of the population—still alive—who were NW, OW, OB_1_, or OB_2_ at different ages for women (a) and men (b).

In a Norwegian cohort of 2-y-olds, the proportion of OW individuals is expected to increase to the age of ≈45 y and stabilize thereafter. The number of OB_1_ and OB_2_ individuals is expected to increase to the age of ≈70–80 y and thereafter decrease. Compared with men, women are expected to stabilize at a lower rate of OW but have higher rates of OB_1_ and OB_2_ (see Figures 4a–b).

### Health Care Costs

For women, estimates from the 2-part model showed average marginal effects associated with OW of €160 (*P* = 0.06) and significant effects at a 5% level for OB_1_ (€631, *P* < 0.001) and OB_2_ (€698, *P* < 0.001) as compared with NW. Among men, there were nonsignificant average marginal effects associated with OW (−€96, *P* = 0.31) and OB_1_ (€182, *P* = 0.15) but a significant effect for OB_2_ (€1456, *P* < 0.001) when compared with NW (see Appendix 4, Table s4 and s5).

**Figures 5:**
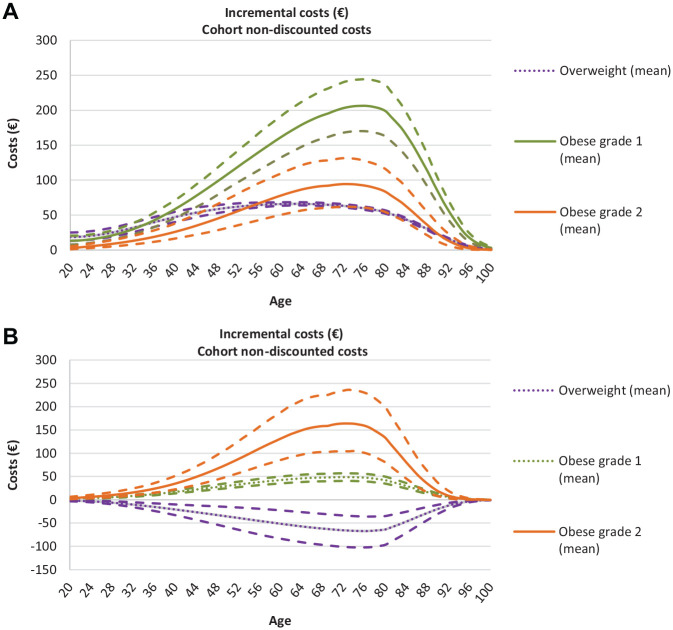
Expected survival-adjusted incremental health care costs per year with 95% confidence bounds as the cohort ages for women (a) and men (b). NW is the reference value (at €0). Average estimates that are dotted indicate that the point estimate from the 2-part model (cost estimate input) was not significant at a 5% level.

Using these significant and nonsignificant cost estimates as input in the model, we simulated expected annual survival-adjusted incremental health care costs per person in the cohort (Figures 5a and b), which combines the cost of actually being NW, OW, OB_1_, or OB_2_ (from the 2-part model) with the probability of becoming NW, OW, OB_1_, or OB_2_ (from the expected prevalences; Figures 4a, b). The expected costs of OB increased with age and peaked late in life. For women, the expected costs of OW peaked at age 60 at €66 (€64–€68). The expected costs for OB_1_ and OB_2_ peaked at age 75 and were higher for OB_1_ (€206, €170–€244) than for OB_2_ (€93, €61–€129) because of the higher probability of becoming OB_1_ than OB_2_ (Figure 4a). For men, the expected costs related to OW were negative (−€67, −€102 to −€36) and reached their peak at the age of 75. The expected costs for OB_1_ and OB_2_ peaked at age 75 and were higher for OB_2_ (€164, €104–€234) than for OB_1_ (€49, €40–€57).

The cumulative expected survival-adjusted incremental health care costs related to obesity (OB_1_ and OB_2_) per person in the cohort over the lifetime were €12,118 (€9495–€15,047) for women and €6646 (€5252 – €10,900) for men; consequently, the expected lifetime health care costs related to obesity for the whole cohort accumulate to ≈€320 million (€251 million–€498 million) for women and ≈€191 million (€150 million–€312 million) for men (Table 2).

**Table 2:** Lifetime Expected Incremental Survival-Adjusted Health Care Costs Per Person for Women and Men, and, the Percentage of Costs that Accumulated Between the Ages 2−30y, 30−60y and 60+y. Numbers are given in EURO (€) and Percentage (%) and are Displayed Both With and Without Discounting at 4%

	Women (*n* = 26,458)	Men (*n* = 28,662)
OW		
Nondiscounted (€: Mean (95%CB))	3534 (3318; 3764)	−2711 (−4319; −1278)
Discounted (€: Mean (95%CB))	487 (435; 549)	−274 (−439; −125)
OB_1_		
Nondiscounted (€: Mean (95%CB))	8439 (7018; 9973)	2124 (1627; 2395)
Discounted (€: Mean (95%CB))	843 (690; 1011)	230 (175; 264)
OB_2_		
Nondiscounted (€: Mean (95%CB))	3679 (2477; 5074)	4522 (3625; 8505)
Discounted (€: Mean (95%CB))	374 (247; 525)	448 (360; 832)
Total costs		
Nondiscounted (€: Mean (95%CB))	15,651 (12,850; 18,835)	3935 (3852; 6762)
Discounted (€: Mean (95%CB))	1705 (1378; 2074)	405 (393; 689)
Age 2−30 y		
Nondiscounted (%)	0.03	0.02
Discounted (%)	0.11	0.07
Age 30−60 y		
Nondiscounted (%)	0.34	0.32
Discounted (%)	0.53	0.5
Age 60+y		
Nondiscounted (%)	0.64	0.66
Discounted (%)	0.36	0.43

### Mortality and Scenario Analyses

On average, obesity shortened the life expectancy of women in the cohort by 1.31 y (1.6%) and men by 1.08 y (1.3%; scenario 1 and 2). The expected impact of OB1 (difference between scenario 1 and 2) was similar between the genders at 0.74 y versus 0.70 y for women and men, respectively. In summary, obesity reduces the life expectancy of the cohort of 55,120 individuals by a total of ≈65,000 life-years.

The average life expectancy for an NW woman/man at age 30 was 83.31/80.31 y and reduced by 1.05/0.65 y, 2.87/2.71 y, and 4.08/4.83 y, if she or he was OW, OB_1_, or OB_2_, respectively. See Table 3.

**Table 3:** Life Expectancy for the Entire Cohort and When Dividing between Women and Men, Years of Life Loss (YLL) Caused by Obesity I (Scenario 1) and Obesity I and II (Scenario 2), and Expected Life Expectancy for Those Who Are NW, OW, OB_1_, or OB_2_ at 30 y^a^

	Women (*n* = 26,458)			Men (*n* = 28,662)		
	Mean	95% CB	Δ	Mean	95% CB	Δ
Base case	82.53	(81.92–83.14)	r.c.	79.31	(78.66–79.77)	r.c.
Scenario 1	83.10	(82.69–83.50)	0.57	79.69	(79.26–80.11)	0.38
Scenario 2	83.84	(83.54–84.08)	1.31	80.39	(80.09–80.69)	1.08
Total life-years loss										
OB_2_ (scenario 1)^b^	15, 081			10, 891		
OB_1_ (scenario 2)^c^	34, 660			30, 954		
Life expectancy (at 30 y)^d^						
Scenario 3 NW	83.31	(82.84–83.75)	r.c.	80.31	(79.84–80.70)	r.c.
Scenario 4 OW	82.26	(81.53–82.97)	1.05	79.66	(78.91–80.15)	0.65
Scenario 5 OB_1_	80.44	(79.27–81.47)	2.87	77.60	(76.14–78.24)	2.71
Scenario 6 OB_2_	79.23	(77.68–80.64)	4.08	75.48	(74.01–76.94)	4.83

a Δ Presented as difference from reference category.

Scenario 1: eliminating obesity II.

Scenario 2: eliminating obesity I and obesity II. r.c. = reference category.

b Estimated by multiplying the expected life-years lost of obesity II for an individual by the entire cohort (i.e., 0.57 × 26,458 = 15,081).

c Estimated by multiplying the expected life-years lost of obesity I and II for an individual by the entire cohort (i.e., 1.31 × 26,458 = 34,660).

d Life expectancy estimated by assuming an individual is NW (scenario 3), OW (scenario 4), OB1 (scenario 5), or OB2 (scenario 6) at age 30 y in the model. In scenarios 3–6, individuals could move between health states according to transition probabilities as used in the MOON model after the age of 30; thus, individuals who were NW at age 30 could progress to OW, OB1, or OB2 throughout their lifetime. Likewise, individuals who were OB2 at age 30 could regress to OB1, OW, and NW throughout their lifetime.

### Model Validation

**Figure 6:**
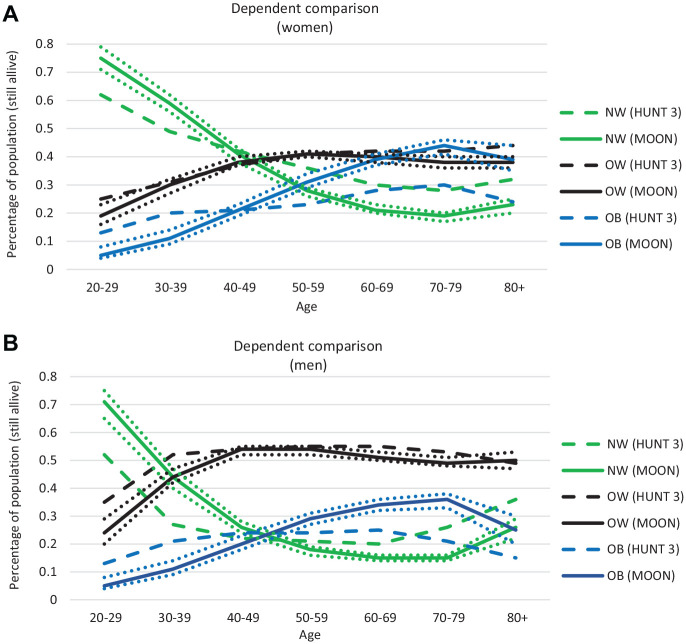
Prevalence of normal weight, overweight, and obesity (grade 1 and 2) as estimated in the HUNT-3 and in the MOON for women (a) and men (b). The straight line gives the mean from the MOON – with 95% CB indicated by the dotted line.

We ensured good face validity through close and continuous cooperation between modelers and clinicians through all steps of the modeling, from conception, statistical analyses and estimation of input parameters, and valuation of the final model. Internal validity was ensured by examining the mathematical calculations used in the model through several steps (for example, by ensuring that the cohorts’ size was constant). We also had a walkthrough of the model with experienced modelers in the field of industrial economics, in which the aim was to search for errors in the logic and mathematical calculations in the model. The external modelers confirmed the models’ internal validity. Figure 6a, b illustrates the external validity (dependent comparison between the model and HUNT 3), which showed that the model predicts a higher prevalence of OB_1_ and OB_2_ but equal levels of OW than what is observed in HUNT-3. The reason is that we are predicting the prevalence of overweight and obesity in the future given that the development in overweight and obesity continues as it has been observed to do between 1980s (HUNT 1) and the 2000s (HUNT 3), in which the prevalence of overweight has stabilized while the prevalence of obesity has increased.^30^ See Supplementary Appendix 6 for more details.

## Discussion

In this article, we describe how we built an obesity-simulation model in the Norwegian context. We validated the model by simulating a birth cohort of 2-y-olds throughout their life. The model predicted that the prevalence of obesity will continue to increase. The expected incremental health care cost per person in the cohort associated with obesity is estimated at €12,118 for women and €6,664 for men, where 65% accumulated at age >60 y. In addition, obesity leads to a YLL of 1.31 y and 1.08 y for woman and men, respectively. Our results indicate that there is a potential for substantial investments to reduce the burden of obesity but that health gains and cost savings might be visible first several decades after the investment.

In Norway, most of the adult population is currently overweight (∼50%) or obese (∼23%).^30,44^ We predict an equal prevalence of overweight but a higher prevalence of obesity in the future, for example, at 41% (overweight) and 31% (obesity) for women and 54% (overweight) and 29% (obesity) for men aged 50 to 59 y. Because we assumed future projections based on recent secular trends (from the 1980s to 2000s), this pattern is intuitive, given the dramatic increase in the prevalence of obesity observed during the past decades.^30,44,45^ Recently, some research has indicated that the prevalence of obesity in northern Europe might be leveling off.^46^ If this is the case, our estimates might be exaggerated. However, our model will be updated continuously—through updated survival analyses or calibration—when new data on the prevalence of obesity in Norway are available (e.g., when HUNT 4 is available).^47^

To the best of our knowledge, no similar predictions have been made for Norway previously. Kelly et al.^48^ projected the prevalence of overweight and obesity in 2030 for individuals aged >20 in countries with established market economies at 58% when assuming stable age-specific prevalence and 66% when assuming a continuation of recent secular trends. Our estimates were at 64% (59%–68%) for women and 70% (65%–76%) for men. In Germany, Sonntag et al.,^23^ who tracked childhood obesity into adulthood, found that 35% of women and 42% of men would be overweight or obese at age 30. Our numbers are similar at 32% (27%–37%) for women and 44% (39%–50%) for men.^23,24^ In the United States, Ward et al.^16^ predicted that most (55%) of today’s children will be obese by the age of 35. These estimates are higher than our estimates of 11% (OB_1_ and OB_2_).^16^ Different data (e.g., the use of cross-sectional versus longitudinal data or the nationality of individuals in the data set) and modeling techniques (e.g., handling of secular trends) influence simulation models both in their ability to produce valid epidemiologic output (e.g., prevalence of obesity) and in their ability to be used in future cost-effectiveness analyses. The nationality of the modeled population (i.e., United States, Norway, and Germany) largely influences the difference in the estimated prevalence of obesity between Ward et al.^16^ Sonntag et al.^23,24^ and the MOON model, since the obesity epidemic in the United States is more mature than in Norway and Germany.^49^ Also, whether and how secular trends are incorporated in models influence the estimated prevalence of obesity, as seen in Kelly et al.,^48^ in which the prevalence of obesity was higher when assuming a continuation of recent secular trends (66%) than when assuming a stable age-specific prevalence (58%).^46^ Whether models are built using cross-sectional or longitudinal data does not necessarily influence whether they produce valid epidemiologic output. Among the above-mentioned papers, Kelly et al. and Sonntag et al. used cross-sectional data, whereas Ward et al. used longitudinal data.^16,23,24,46^ Despite this, our estimated prevalence is more similar to Sonntag et al. and to Kelly et al. than to Ward et al. However, models built using longitudinal data more appropriately capture individuals’ true weight fluctuation and, consequently, the true transition between health states. These models will therefore most likely give more precise estimates in future cost-effectiveness analyses. A possible weakness when using longitudinal data is the potential of increasing selection bias, such as selective survival bias (e.g., by including only individuals from HUNT surviving for at least 1 decade) and healthy volunteer bias (e.g., by including only individuals who choose to participate in HUNT 2 times).^50^ However, this does not seem to influence the prevalence of obesity in the MOON model when comparing it to models using cross-sectional data, and we think that the positive sides of using longitudinal data outweigh the negative sides.

There is wide consensus that obesity leads to increased health care costs,^51^ and the level of costs we estimate concurs with previous research.^40,51–56^ In addition, the patterns we identified align with previous research: that costs peak late in life (at 60–70 y old)^23,40,54^ and are higher among women than men.^23,53–55^ Generally, cost estimates vary substantially between studies.^53^ Since different studies use different data sources, it is hard to disentangle how much of this variation is caused by differences in the analytical frameworks applied or the populations under analysis. For example, reversed causality (e.g., the fact that disease decreases body weight) is likely a source of bias within obesity and is handled differently between studies.^53^ Cawley et al.^54^ and Dixton et al.,^56^ who used weight of a biological relative and genetic variation in instrumental variable analyses to estimate the causal inferences between obesity and health care costs, found that cost estimates were substantially higher (>50%) in their analyses than in multivariable regression analyses. Consequently, our cost estimates might be too low, but they are, at the moment, the most accurate cost estimates made for Norway. Because of the simple nature of our model, more precise cost estimates, if made available, can easily be used as input in future versions of the model.

We find that overweight and obesity will lead to an average expected YLL of 1.31 and 1.08 y for women and men in the cohort, respectively. To the best of our knowledge, no similar numbers have been previously estimated for obesity, but as a comparison, the average YLL of socioeconomic inequality is estimated at 14.4 mo (1.2 y).^57^ We also estimated the YLL for 30-y-old women and men conditional on their weight category (Table 3), and our findings are in line with previous research: Fontaine et al.^58^ estimated that the YLL for 30-y-old white women and men was 0.5 y for a BMI of 30 and increased to 4 y (women) and 5 y (men) if their BMI was 40. Grover et al.^59^ estimated the YLL for very obese men and women to be 8.4 y and 6.9 y (in those aged 20–39 y), respectively, whereas Finkelstein et al.^60^ estimated that the YLL loss for 40-y-old white women and men was 1 y for those with OB_1_ and 4 y (women) and 5 y (men) for those with OB_2_.

This article, and the MOON model, has several strengths. To the best of our knowledge, this is the first obesity-focused DSM built for a Norway context. Because our model shows valid results, it can be used in future economic evaluations. Obesity is a complex and multifactorial disease with genetic, behavioral, socioeconomic, and environmental origins; changes will most likely happen during the lifetime of a child cohort that we are not be able to simulate.^7,61^ This should be carefully considered when evaluating interventions in the future, by, in accordance with Squires et al.,^62^ putting strong effort into understanding the current decision-making process, identifying and involving relevant stakeholders, understanding the relevant problem, and consequently developing and/or justifying the decision model being used to make estimates of the cost-effectiveness. As an example, the ACE-Obesity program in Australia involved a large group of stakeholders who identified relevant interventions. The effect of these interventions was estimated as the expected change on BMI development, preferably directly from clinical trials, and when this was not available, through estimates of the relationship between energy imbalances (from interventions) on change in body weight.^22,63^ They used simulation models, similar to the MOON, as a basis to extrapolate results in a longer time perspective.

Next, for the purpose of transparency, we aimed at building a simple and transparent model that is easy to understand and use.^64,65^ Although simplicity is important, the model also needs to be sufficiently complex to represent the complexity of the modeled problem and to exhibit satisfactory validity.^64^ The quality of data regarding BMI across Europe is generally regarded as poor.^66^ We are among the first to have access to longitudinal data on large cohorts (*n* = 99,348) covering the ages 2 to 102 y. The use of real-world data provides us a unique opportunity to contribute with new knowledge regarding obesity, such as the level of transition between health states, and the expected prevalence, health care costs, and mortality of obesity, all depending on gender and age. This knowledge will in itself contribute to the obesity literature. For example, although the prevalence of obesity increases steadily throughout a cohort’s lifetime, there was, in fact, a large proportion of individuals (particularly children) who moved between health states annually. In many children, these movements likely reflect natural fluctuations related to growth spurts, rather than true overweight. This is not possible to detect if using cross-sectional data^23,24^ but should be considered when introducing new interventions.

The simple but realistic nature of the MOON model can also enable researchers from other countries to duplicate and calibrate the MOON to their settings if they have access to proper targets from cross-sectional data (e.g., epidemiologic data on the prevalence of obesity).^47^

The MOON also has several shortcomings worth mentioning. We used a health care perspective, which is recommended in Norway.^67^ However, obesity affects individuals not only through disease but also through factors such as stigma and bullying, thus influencing school and work participation.^24,51,52,68^ Using a health care perspective might therefore ignore important societal (short-time) effects of preventing obesity. Because of the simple but still flexible framework used in the MOON, our model can easily be extended to include the societal perspective in the future by estimating a variety of consequences (e.g., dropping out of school) depending on BMI category and age.

The development of obesity in the population depends on both socioeconomic status (SES) and living situation (urban or rural living).^69^ We did not include these factors in our model—primarily because they were not available in the childhood cohort—and therefore cannot differentiate between populations based on SES and living situation. In the future, we can extend the model to differentiate between populations based on SES or living situation, either by using estimates of relative risks of SES and living situation on transition probabilities, or—if data are available—by including these factors in survival analyses when predicting transition probabilities.

In Norway, the life expectancy at birth in 2018 was 84.49 y for women and 81.00 y for men.^70^ These numbers are higher than the life expectancy estimated for the cohort in the model (82.53 y for women and 79.31 y for men; see Table 1). This discrepancy is primarily caused by the modeled high rates of obesity in the future and thus also higher rates of mortality. The modeled life expectancy is similar to Norwegian estimates in 2009 (83.05 y for women and 78.6 y for men). In the future, the life expectancy is expected to increase even more (i.e., to 88.2 y for women and 85.8 y for men in 2050). An important question is whether the incremental life expectancy between normal-weight individuals and those who are overweight, obese grade 1 or obese grade 2, will change as the life expectancy of the population changes. The life expectancy of a population depends on factors such as health, living conditions, quality of the health care system, medical development, and changes in the population’s lifestyle and quality of life.^71^ It is difficult to predict how these factors will change and be different in the future among those with different weight status. However, to ensure that the model is as realistic and up to date as possible when it is used in future cost-effectiveness analyses, it will be updated with the most recent numbers from life tables and the most recent (and reliable) numbers on hazard ratios between normal weight, overweight, obese grade 1, and obese grade 2.

Finally, obesity treatment is primarily provided to people with severe obesity.^72^ For the severely obese, a stabilization or small reduction in weight can be considered a success.^73^ These patients would not necessarily cross over from OB_2_ to OB_1_ in the MOON (see Figure 1); consequently, these treatment effects would not be captured by the model. However, estimating these effects is important, because the risk of adverse health outcomes increases exponentially with weight gain. A stabilization or small reduction in BMI could in fact have a large effect on the individual’s morbidity, mortality, and health care costs.^50^ A treatment modality of the MOON model, including only the severely obese with a BMI output on a continuous scale (similar to Ward et al.), should be pursued in the future.^16^

## Conclusion

In this article, we describe how we built a data-heavy, valid, and flexible obesity-focused disease-simulation model in the Norwegian context (the MOON model). Through simulation for a Norwegian birth cohort (*n* = 55,120), the model predicts a high prevalence of obesity, leading to an incremental health care cost of €511 million over the lifetime while causing a loss of ≈65,000 life-years. The results underscore the urgent need to implement preventive and treatment interventions and illustrates potential cost savings associated with effective interventions. In the future, the MOON model can be used as a tool to inform policy makers regarding the expected long-term effect and cost-effectiveness of different sex- and age-specific interventions.

## Supplemental Material

sj-docx-1-mdm-10.1177_0272989X20971589 – Supplemental material for Modeling Obesity in Norway (The MOON Study): A Decision-Analytic Approach—Prevalence, Costs, and Years of Life Lost Click here for additional data file. Supplemental material, sj-docx-1-mdm-10.1177_0272989X20971589 for Modeling Obesity in Norway (The MOON Study): A Decision-Analytic Approach—Prevalence, Costs, and Years of Life Lost by Gudrun M. W. Bjørnelv, Vidar Halsteinli, Bård E. Kulseng, Diana Sonntag and Rønnaug A. Ødegaard in Medical Decision Making

sj-docx-2-mdm-10.1177_0272989X20971589 – Supplemental material for Modeling Obesity in Norway (The MOON Study): A Decision-Analytic Approach—Prevalence, Costs, and Years of Life Lost Click here for additional data file. Supplemental material, sj-docx-2-mdm-10.1177_0272989X20971589 for Modeling Obesity in Norway (The MOON Study): A Decision-Analytic Approach—Prevalence, Costs, and Years of Life Lost by Gudrun M. W. Bjørnelv, Vidar Halsteinli, Bård E. Kulseng, Diana Sonntag and Rønnaug A. Ødegaard in Medical Decision Making
