# Supplementary material for: Modeling Obesity in Norway (The MOON Study): A Decision-Analytic Approach—Prevalence, Costs, and Years of Life Lost
Source: Med Decis Making. 2020 Nov 30;41(1):21–36. doi: 10.1177/0272989X20971589 (PMC7783689; doi:10.1177/0272989X20971589)
Supplement: sj-docx-2-mdm-10.1177_0272989X20971589 – Supplemental material for Modeling Obesity in Norway (The MOON Study): A Decision-Analytic Approach—Prevalence, Costs, and Years of Life Lost [file sj-docx-2-mdm-10.1177_0272989X20971589.docx]

**Supplementary Appendix**

**MOdeling Obesity in Norway (the MOON-study): a decision-analytic approach**

Prevalence, costs and years of life lost.

PhD Gudrun M.W. Bjørnelv, PhD Vidar Halsteinli, Professor MD Bård E. Kulseng, Dr. rer. pol. habil. Diana Sonntag, and PhD MD Rønnaug A. Ødegaard.

**Table of content**

Supplementary Appendix 1 – Schematic of data,,,,,,,,,,,,,,,,,,,,,,,,,,,,,,,,,,,,,,,,,,,,,,,,,,,,,,,,,,,,,,,,,,,, 2

Supplementary Appendix 2 – Interpolation ,,,,,,,,,,,,,,,,,,,,,,,,,,,,,,,,,,,,,,,,,,,,,,,,,,,,,,,, 4

Supplementary Appendix 3 – Mortality rates ,,,,,,,,,,,,,,,,,,,,,,,,,,,,,,,,,,,,,,,,,,,,,,,,,,,,,,,, 5 Supplementary Appendix 4 – Cost estimates,,,,,,,,,,,,,,,,,,,,,,,,,,,,,,,,,,,,,,,,,,,,,,,,,,,,,,,,,,,,,,,,,,,,,,,,, 6

Supplementary Appendix 5 – Transition probabilities,,,,,,,,,,,,,,,,,,,,,,,,,,,,,,,,,,,,,,,,,,,,,,,,,,,,,, 24

Supplementary Appendix 6 - Validation of the model,,,,,,,,,,,,,,,,,,,,,,,,,,,,,,,,,,,,,,,,,,,,,,,,,,,,,,, 32

*Face validity,,,,,,,,,,,,,,,,,,,,,,,,,,,,,,,,,,,,,,,,,,,,,,,,,,,,,,,,,,,,,,,,,,,,,,,,,,,,,,,,,,,,,,,,,,,,,,,,,,,,,,,,,,,,,,,,,,,,,,,,,,,,,,,,,,,,,,,,,,,,,,,,,, 32*

*Internal validity,,,,,,,,,,,,,,,,,,,,,,,,,,,,,,,,,,,,,,,,,,,,,,,,,,,,,,,,,,,,,,,,,,,,,,,,,,,,,,,,,,,,,,,,,,,,,,,,,,,,,,,,,,,,,,,,,,,,,,,,,,,,,,,,,,,,,,,,,,, 32*

*External validity, dependent comparison 1,,,,,,,,,,,,,,,,,,,,,,,,,,,,,,,,,,,,,,,,,,,,,,,,,,,,,,,,,,,,,,,,,,,,,,,,,,,,,,,,,,,,,,, 33*

*External validity, dependent comparison 2,,,,,,,,,,,,,,,,,,,,,,,,,,,,,,,,,,,,,,,,,,,,,,,,,,,,,,,,,,,,,,,,,,,,,,,,,,,,,,,,,,,,,,, 35*

References,,,,,,,,,,,,,,,,,,,,,,,,,,,,,,,,,,,,,,,,,,,,,,,,,,,,,,,,,,,,,,,,,,,,,,,,,,,,,,,,,,,,,,,,,,,,,,,,,,,,,,,,,,,,,,,,,,,,,,,,,,,,,,,,,,,,, 37

# Supplementary Appendix 1 - Schematic and explanation of the data used to estimate transition probabilities in the Markov Model.


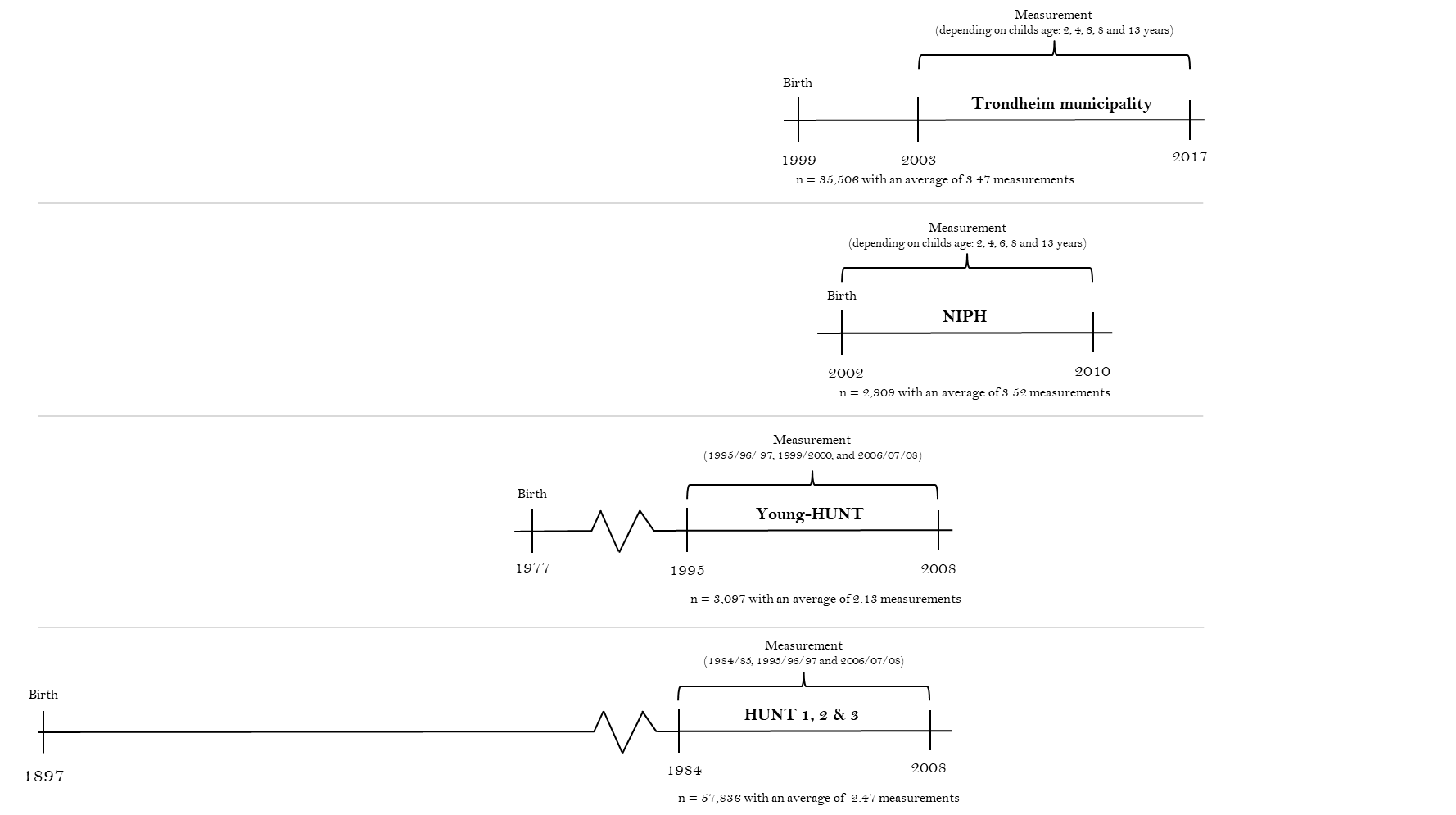


Figure S1: schematic of data used to estimate transition probabilities. Participants were restricted to those with a minimum of 2 observations

**Selection for and participation in the adult dataset**

In total, we had information on the BMI of 103,967 participants from the adult HUNT 1, 2 and 3 surveys. Of these, 25,801, 8,957 and 11,373 only participated in HUNT 1, HUNT 2 or HUNT 3, respectively, and were not included in the analyses. The distribution of participation for the 57,836 individuals with two or more observations who were included in the adult dataset can be seen in Table s1:

Tabell S1: Distribution of participation for the 57,836 individuals who were included in the adult dataset (HUNT 1, 2 and 3).

| **Survey wave** | | |  |
| --- | --- | --- | --- |
| **HUNT 1** | **HUNT 2** | **HUNT 3** | **n** |
| x | x | x | 27,389 |
| x | x |  | 18,799 |
| x |  | x | 2,340 |
|  | x | x | 9,308 |

**Selection for and participation in the adolescent dataset**

In total, we had information from 16,084 individuals from Young HUNT 1, 2 and/or 3 with measurement on height and weight. When linked to HUNT 3, 12,974 individuals only had one observation while 13 had illogic observations; these were excluded. The distribution of participation for the 3,097 individuals with two or more observations who were included in the adolescent dataset can be seen in Table s2:

**Tabell** **S2: Distribution of participation for the 3,097 individuals who were included in the adolescent dataset (Young-HUNT 1, 2 and 3 and HUNT 3).**

| Survey wave | | | |  |
| --- | --- | --- | --- | --- |
| YH1 | YH2 | YH3 | aH4 | n |
| x | x |  |  | 1,233 |
| x |  |  | x | 1,417 |
|  | x |  | x | 11 |
|  |  | x | x | 18 |
| x | x |  | x | 418 |

# Supplementary Appendix 2 – Interpolation

HUNT 1, 2 and 3 were performed in three waves in 1984-1986, 1995-1997 and 2006-2008. Without interpolation, the survival analyses would not capture changes happening until 10 years after the first observations, thus inducing a false low transition in the first observation period in all analyses. We interpolated values by estimating the height and weight between measurement points. For the children cohort (T-MU and COIS), data were gathered at sufficiently close time gaps, and no interpolation was performed. In the adolescent populations (Young-Hunt 1 and 2 and adult HUNT 3) and the adult populations (HUNT 1, 2 and 3), we interpolated values at a yearly basis between the measurements. We assumed a linear increase in height until the age of 18, when height was assumed to be constant, and a linear increase in weight between all measurements (both adolescents and adults).

# Supplementary Appendix 3 – Mortality rates

We estimated age and gender-specific mortality rates from Norwegian life tables at Statistics Norway (SSB), for numbers based on the year 2017. We multiplied the mortality rate for those overweight, obese grade 1 and obese grade 2 with hazard ratios estimated from the Global BMI Mortality Collaboration (1). Normal weight was the reference group in Table S3.The Global BMI Mortality Collaboration made their estimates for the age groups 35-49, 50-69 and 70-89, using 10,625,411 participants from 239 prospective studies. We used estimates from their primary analyses, which were performed to limit residual confounding and reversed causality. These analyses were restricted to never-smokers without specific known chronic diseases at baseline (e.g., cardiovascular disease, cancer, or respiratory disease) and five years of follow up was omitted. We used estimates they made for Europe.

Table S3: Hazard ratio for the overweight, obese grade 1 and obese grade 2, with 95% confidence intervals (used in the probabilistic sensitivity analyses (PSAs)). Numbers taken from the Global BMI Mortality Collaboration (1) eTable 11, where values were stratified by region (we used European estimates).

|  | Overweight | | | Obese grade 1 | | | Obese grade 2ᴬ | | |
| --- | --- | --- | --- | --- | --- | --- | --- | --- | --- |
| Age groups | HR | 95% CI | | HR | 95% CI | | HR | 95% CI | |
| 35 – 49 | 1.17 | 1.15 | 1.20 | 1.90 | 1.72 | 2.09 | 3.48 | 2.93 | 4.12 |
| 50 – 69 | 1.11 | 1.07 | 1.15 | 1.60 | 1.51 | 1.70 | 2.59 | 2.36 | 2.83 |
| 70 – 89 | 0.98 | 0.93 | 1.02 | 1.12 | 1.03 | 1.21 | 1.63 | 1.27 | 2,10 |

ᴬ **In HUNT 3 2,650 individuals were obese grade 2. Of these, 537 (20%) had a BMI > 40. We therefore estimated the HR of being in Obese grade 2 as 80% of the hazard rate for those with a BMI between 35 and 40 and 20% of the hazard rate for those with a BMI above 40 from the Global BMI Mortality For example, in the age group 35-49: HR (average) = 3.01*0.8 + 5.34*0.2=3,476. HR 95% CI (lower bound) = 2,62*0,8+4,2*0,2 = 2,936. HR 95% CI (upper bound) = 3,46*0,8+6,78*0,2 = 4,124.**

# Supplementary Appendix 4 – Cost estimates

*The Norwegian healthcare system and service categories*

In Norway, health care services are divided into specialised- and primary health care, both are an integral part of the welfare system and are provided in a predominantly public and tax based health care system (2). The present study was conducted from a health care perspective and major specialised care service categories were included, namely hospital services. In addition, we included use of general practitioners (primary care). Costs were estimated by multiplying service volumes by a unit cost and summarizing over service categories. The included service categories and the accompanying source of unit costs are listed in Table S4.

Table s4: Type of service and sources used to estimates costs.

| **Service category** | **Service unit** | **Source - unit cost estimation** |
| --- | --- | --- |
| **Hospital (somatic care)** | |  |
| Inpatient care | Stays | DRG-weights* and national unit cost (2009) (3) |
| Day-care | Days/treatments | DRG-weights* and national unit cost (2009) (3) |
| Outpatient care | Visits | DRG-weights* and national unit cost (2009) (3) |
|  |  |  |
| **Hospital (psyciatric care)** | |  |
| Inpatient care | Days | Published unit cost, SINTEF-report (4) |
| Day-care | Days/treatments | Published unit cost, SINTEF-report (4) |
| Outpatient care | Visits | Published unit cost, SINTEF-report (4) |
|  |  |  |
| **Private specialist** | |  |
| Outpatient care | Visits | Calculation based on DRG-weights* and national unit costs (using the same cost depending on the ICD-10 code of the patient) |
|  |  |  |
| **Hospital (drug and alcohol treatment)** | |  |
| Inpatient care | Days | Published unit cost, The Norwegian Directorate of Health (5). |
| Outpatient care | Visits | Published unit cost, The Norwegian Directorate of Health (5). |
|  |  |  |
| **General practitioner** | Contact ** | GP-tariffs (based on claims) and patient co-payments * 2 (to estimate total costs) (ref) |

*** DRG-weights vary depending on the resources required for a treatment belonging to a specific DRG-category. Unit cost in 2009 for 1 DRG-point = EURO 4,024 (for inpatient and day care) and EURO 122 (for outpatient care).**

**** GP contacts include both standard in-office visits and a range of supplementary services e.g. laboratory tests and telephone contact.**

*Hospital - somatic care*

Use of somatic hospital inpatient- , day- and outpatient care episodes were identified by linking the n=49714 HUNT-3 individuals, who were still alive and living in Norway January 1^st^ 2010, to data from the Norwegian Patient Register (NPR) for 2009. Costs were calculated from cost-weights using diagnosis-related groups (DRG); in Norway, all somatic hospitals electronically report patient activity to NPR. These reports include information regarding the patients diagnoses (ICD-10 codes) and which procedures that were used to treat the patient. This information forms the basis for grouping all patient episodes into DRG’s which in turn forms the basis for Activity-Based-Funding. In this way, each patient episode is grouped into one of approximately 900 DRGs, and each DRG holds an associated cost-weight that can be used to estimate the average cost of an inpatient, day- or outpatient care episode. The DRG-costs are expected to cover average costs of a treatment in Norway. For outpatient visits, a patient co-payment has to be added (fixed to EURO 35 per visit in 2009) (3).

*Hospital – psychiatric care*

Use of psychiatric hospital inpatient- , day- and outpatient care were identified from NPR in the same manner as somatic care. However, in 2009 psychiatric care was not financed through Activity Based Funding and consequently, we could not use information from DRGs’ to estimate these costs. SINTEF have made average cost estimates for inpatient and outpatient care that we used. For inpatient stays, cost were calculated by multiplying the length of stay (in days) by and estimate of the average cost per day (€815). For day-care, we multiplied each day by half of the cost per day of inpatient care (€407,5). All outpatient visits were multiplied by an average unit cost estimated at €247 (4).

*Private specialist on contract*

Use of private specialists on contract (publicly funded) were identified from NPR in the same manner as somatic care. However, in 2009 private information regarding the DRG of treatment provided by specialists on contract were not available in NPR, and consequently, we could not use information from DRGs’ to estimate these costs. We assumed that an outpatient visit at a private specialist had the same average cost as similar visits in a publicly hospital, differentiating on ‘letter’ in the ICD-10 system. For example, patients were assumed to be similar if they were diagnosed with ICD-10 codes A00-B99, C00 – C99, D00-D99 etc.

*Hospital – multi-disciplinary drug and alcohol treatment*

Use of hospital drug- and alcohol treatments were identified from NPR in the same manner as somatic care. However, in 2009 hospital drug- and alcohol treatment was not financed through Activity Based Funding and consequently, we could not use information from DRGs’ to estimate these costs. For inpatient stays cost were calculated by multiplying length of stay by average cost per day (€700), as reported by The Norwegian Directorate of Health. All outpatient visits were multiplied by an average unit cost (€389) (5).

*General practitioners*

Use of general practitioners (GPs) were identified by linking the n=49 417 HUNT-3 individuals to data from the Health Reimbursement Register (KUHR). This register holds information on claims from GPs and patient-copayments (6). We received information from KUHR on all claims from GP consultations/visits that occurred during 2009, the corresponding reimbursement tariff, and the out-of-pocket payment paid per visit. The Norwegian Directorate of Health has previously estimated that the claim reimbursement and patient co-payment covers approximately 50% of the total cost of care of a GP visit. We therefore divided the claim and patient-copayment by 0.5 to estimate total costs of care (7).

Table S5 presents descriptive statistics for health service utilization volumes and the calculated costs in 2009. Among the n=49 714 individuals, 12% had received hospital somatic inpatient care, while the accompanying costs accounted for 50% of the total calculated costs that year. A total of 87% had at least one GP visit, while the GP costs in total accounted for 16% of the calculated costs. In total, somatic hospital services accounted for 69% of the calculated costs, psychiatric hospital services 11%, private specialists 3%, hospital drug and alcohol treatment 1% and GPs 16%.

**Table s5: Use of healthcare services among patients in HUNT 3 who were still alive and living in Norway January 1st 2010 (n=49,714)**

| **Service category** | **No. users** | **Share users** | **Cost per user** | **Total costs** | **% costs per service category** |
| --- | --- | --- | --- | --- | --- |
| **Hospital (somatic care)** | |  |  |  |  |
| Inpatient care | 5 732 | (0.12) | 7 624 | 43 702 880 | 50.5 % |
| Day-care | 2 794 | (0.06) | 2 770 | 7 739 369 | 8.9 % |
| Outpatient care | 18 898 | (0.38) | 437 | 8 257 083 | 9.5 % |
|  |  |  |  |  |  |
| **Hospital (psyciatric care)** | |  |  |  |  |
| Inpatient care | 214 | (0.00) | 29 258 | 6 261 145 | 7.2 % |
| Day-care | 1 | (0.00) | 4 893 | 4 893 | 0.0 % |
| Outpatient care | 1 226 | (0.02) | 2 429 | 2 977 766 | 3.4 % |
|  |  |  |  |  |  |
| **Private specialist** |  |  |  |  |  |
| Outpatient care | 5 273 | (0.11) | 523 | 2 758 821 | 3.2 % |
|  |  |  |  |  |  |
| **Hospital (drug and alcohol treatment)** | |  |  |  |  |
| Inpatient care | 21 | (0.00) | 45 593 | 957 444 | 1.1 % |
| Outpatient care | 68 | (0.00) | 4 131 | 280 936 | 0.3 % |
|  |  |  |  |  |  |
| **General practitioner** | 42 852 | (0.86) | 318 | 13 631 698 | 15.7 % |

# Results from two-part model

Table s6: Output from the two part model, with the logistic regression and generalized linear model at the left hand side, and average marginal effects (€) at the right hand side. Numbers presented for women.

|  | **Two part model** | | | | | | | | |  | **Average marginal effect** | | | |
| --- | --- | --- | --- | --- | --- | --- | --- | --- | --- | --- | --- | --- | --- | --- |
|  | Logistic regression | | | |  | Generalized linear model | | | |  |  |  |  |  |
|  | **Coefficient** | **SE** | **95% CI** | |  | **Coefficient** | **SE** | **95% CI** | |  | **Coefficient** | **SE** | **95% CI** | |
| Age (continous) | *0,02* | *0,00* | *(0,02* | *0,02)* |  | *0,01* | *0,00* | *(0,01* | *0,02)* |  | *29* | *3* | *(23* | *35)* |
|  |  |  |  |  |  |  |  |  |  |  |  |  |  |  |
| BMI-category |  |  |  |  |  |  |  |  |  |  |  |  |  |  |
| *Normal weight* | *-* | *-* | *-* | *-* |  | *-* | *-* | *-* | *-* |  | *-* | *-* | *-* | *-* |
| *Overweight* | *0,29* | *0,05* | *(0,19* | *0,39)* |  | *0,07* | *0,05* | *(-0,02* | *0,17)* |  | *160* | *83* | *(-4* | *323)* |
| *Obese grade 1* | *0,44* | *0,07* | *(0,30* | *0,58)* |  | *0,31* | *0,06* | *(0,18* | *0,43)* |  | *631* | *127* | *(381* | *880)* |
| *Obese grade 2* | *0,97* | *0,13* | *(0,71* | *1,23)* |  | *0,31* | *0,09* | *(0,14* | *0,49)* |  | *698* | *193* | *(319* | *1 077)* |
|  |  |  |  |  |  |  |  |  |  |  |  |  |  |  |
| Smokin status |  |  |  |  |  |  |  |  |  |  |  |  |  |  |
| *Never smoker* | *-* | *-* | *-* | *-* |  | *-* | *-* | *-* | *-* |  | *-* | *-* | *-* | *-* |
| *Previous smpker* | *0,25* | *0,06* | *(0,14* | *0,36)* |  | *0,14* | *0,05* | *(0,04* | *0,24)* |  | *270* | *90* | *(94* | *446)* |
| *Daily smoker* | *0,32* | *0,07* | *(0,19* | *0,45)* |  | *0,33* | *0,06* | *(0,21* | *0,44)* |  | *666* | *124* | *(423* | *910)* |
| *Occational smoker* | *0,25* | *0,10* | *(0,06* | *0,44)* |  | *-0,08* | *0,09* | *(-0,26* | *0,09)* |  | *-98* | *136* | *(-364* | *168)* |
|  |  |  |  |  |  |  |  |  |  |  |  |  |  |  |
| Marital status |  |  |  |  |  |  |  |  |  |  |  |  |  |  |
| *Married* | *-* | *-* | *-* | *-* |  | *-* | *-* | *-* | *-* |  | *-* | *-* | *-* | *-* |
| *Never married* | *0,30* | *0,07* | *(0,17* | *0,42)* |  | *0,23* | *0,06* | *(0,11* | *0,36)* |  | *467* | *128* | *(217* | *717)* |
| *Widow/ widower* | *0,47* | *0,11* | *(0,25* | *0,68)* |  | *0,27* | *0,08* | *(0,12* | *0,41)* |  | *553* | *158* | *(243* | *863)* |
| *Divorce/ separated* | *0,31* | *0,08* | *(0,14* | *0,47)* |  | *0,13* | *0,07* | *(-0,01* | *0,27)* |  | *269* | *132* | *(10* | *528)* |
|  |  |  |  |  |  |  |  |  |  |  |  |  |  |  |
| *Constant* | *1,03* | *0,10* | *(0,82* | *1,23)* |  | *8,61* | *0,10* | *(8,42* | *8,80)* |  | - | - | - | - |

Table s7: Output from the two part model, with the logistic regression and generalized linear model at the left hand side, and average marginal effects (EURO, €) at the right hand side. Numbers presented for men.

|  | **Two part model** | | | | | | | | |  | **Average marginal effect** | | | |
| --- | --- | --- | --- | --- | --- | --- | --- | --- | --- | --- | --- | --- | --- | --- |
|  | Logistic regression | | | |  | Generalized linear model | | | |  |  |  |  |  |
|  | **Coefficient** | **SE** | **95% CI** | |  | **Coefficient** | **SE** | **95% CI** | |  | **Coefficient** | **SE** | **95% CI** | |
| Age (continous) | *0,04* | *0,00* | *(0,04* | *0,04)* |  | *0,03* | *0,00* | *(0,03* | *0,03)* |  | *57* | *4* | *(49* | *65)* |
|  |  |  |  |  |  |  |  |  |  |  |  |  |  |  |
| BMI-category |  |  |  |  |  |  |  |  |  |  |  |  |  |  |
| *Normal weight* | *-* | *-* | *-* | *-* |  | *-* | *-* | *-* | *-* |  | *-* | *-* | *-* | *-* |
| *Overweight* | *0,10* | *0,04* | *(0,02* | *0,19)* |  | *-0,08* | *0,06* | *(-0,20* | *0,04)* |  | *-96* | *96* | *(-284* | *93)* |
| *Obese grade 1* | *0,48* | *0,06* | *(0,36* | *0,59)* |  | *0,05* | *0,07* | *(-0,09* | *0,20)* |  | *182* | *127* | *(-67* | *432)* |
| *Obese grade 2* | *0,96* | *0,13* | *(0,70* | *1,21)* |  | *0,55* | *0,13* | *(0,30* | *0,81)* |  | *1456* | *376* | *(720* | *2 192)* |
|  |  |  |  |  |  |  |  |  |  |  |  |  |  |  |
| Smokin status |  |  |  |  |  |  |  |  |  |  |  |  |  |  |
| *Never smoker* | *-* | *-* | *-* | *-* |  | *-* | *-* | *-* | *-* |  | *-* | *-* | *-* | *-* |
| *Previous smpker* | *0,39* | *0,05* | *(0,30* | *0,48)* |  | *0,27* | *0,06* | *(0,16* | *0,38)* |  | *465* | *85* | *(298* | *632)* |
| *Daily smoker* | *0,29* | *0,06* | *(0,18* | *0,40)* |  | *0,63* | *0,07* | *(0,48* | *0,77)* |  | *1169* | *160* | *(854* | *1 483)* |
| *Occational smoker* | *0,15* | *0,07* | *(0,02* | *0,28)* |  | *0,28* | *0,10* | *(0,09* | *0,47)* |  | *433* | *154* | *(132* | *734)* |
|  |  |  |  |  |  |  |  |  |  |  |  |  |  |  |
| Marital status |  |  |  |  |  |  |  |  |  |  |  |  |  |  |
| *Married* | *-* | *-* | *-* | *-* |  | *-* | *-* | *-* | *-* |  | *-* | *-* | *-* | *-* |
| *Never married* | *0,09* | *0,05* | *(-0,01* | *0,18)* |  | *0,23* | *0,07* | *(0,09* | *0,37)* |  | *414* | *132* | *(156* | *673)* |
| *Widow/ widower* | *0,54* | *0,18* | *(0,18* | *0,90)* |  | *0,13* | *0,13* | *(-0,13* | *0,39)* |  | *296* | *239* | *(173* | *765)* |
| *Divorce/ separated* | *0,08* | *0,07* | *(-0,05* | *0,22)* |  | *0,26* | *0,08* | *(0,09* | *0,43)* |  | *468* | *164* | *(147* | *789)* |
|  |  |  |  |  |  |  |  |  |  |  |  |  |  |  |
| *Constant* | *-0,81* | *0,09* | *(-0,98* | *-0,63)* |  | *7,67* | *0,13* | *(7,43* | *7,92)* |  | - | - | - | - |

Table S8-S15 display predicted absolute values (assuming average effects) of being normal weight, overweight, obese grade 1 and obese grade 2, at representative values for age for females (S 8-11) and males (S 12-15).

Table s8: Predicted costs (€) for the normal weight estimated as the average effects at representative values of age for females.

| Age | Normal weight | **SE** | Lower 95% bound | Upper 95% bond |
| --- | --- | --- | --- | --- |
| 20 | 883 | 54 | 777 | 990 |
| 21 | 899 | 54 | 793 | 1005 |
| 22 | 915 | 54 | 809 | 1021 |
| 23 | 931 | 54 | 826 | 1036 |
| 24 | 948 | 53 | 843 | 1052 |
| 25 | 964 | 53 | 861 | 1068 |
| 26 | 981 | 53 | 878 | 1084 |
| 27 | 999 | 52 | 896 | 1101 |
| 28 | 1016 | 52 | 914 | 1118 |
| 29 | 1034 | 52 | 933 | 1135 |
| 30 | 1052 | 51 | 951 | 1152 |
| 31 | 1070 | 51 | 970 | 1170 |
| 32 | 1089 | 51 | 990 | 1188 |
| 33 | 1108 | 50 | 1009 | 1206 |
| 34 | 1127 | 50 | 1029 | 1225 |
| 35 | 1146 | 50 | 1049 | 1244 |
| 36 | 1166 | 49 | 1069 | 1263 |
| 37 | 1186 | 49 | 1090 | 1282 |
| 38 | 1206 | 49 | 1110 | 1302 |
| 39 | 1227 | 49 | 1131 | 1323 |
| 40 | 1248 | 49 | 1152 | 1343 |
| 41 | 1269 | 49 | 1174 | 1365 |
| 42 | 1291 | 49 | 1195 | 1386 |
| 43 | 1313 | 49 | 1217 | 1408 |
| 44 | 1335 | 49 | 1239 | 1431 |
| 45 | 1357 | 49 | 1261 | 1454 |
| 46 | 1380 | 50 | 1283 | 1477 |
| 47 | 1403 | 50 | 1305 | 1501 |
| 48 | 1427 | 51 | 1328 | 1526 |
| 49 | 1451 | 51 | 1350 | 1551 |
| 50 | 1475 | 52 | 1373 | 1577 |
| 51 | 1500 | 53 | 1396 | 1604 |
| 52 | 1525 | 54 | 1419 | 1631 |
| 53 | 1550 | 55 | 1442 | 1658 |
| 54 | 1576 | 57 | 1465 | 1687 |
| 55 | 1602 | 58 | 1488 | 1716 |
| 56 | 1629 | 60 | 1512 | 1746 |
| 57 | 1656 | 61 | 1535 | 1776 |
| 58 | 1683 | 63 | 1559 | 1807 |
| 59 | 1711 | 66 | 1582 | 1839 |
| 60 | 1739 | 68 | 1606 | 1872 |
| 61 | 1767 | 70 | 1630 | 1905 |
| 62 | 1796 | 73 | 1654 | 1939 |
| 63 | 1826 | 75 | 1678 | 1974 |
| 64 | 1856 | 78 | 1702 | 2009 |
| 65 | 1886 | 81 | 1726 | 2045 |
| 66 | 1917 | 85 | 1751 | 2082 |
| 67 | 1948 | 88 | 1775 | 2120 |
| 68 | 1979 | 91 | 1800 | 2159 |
| 69 | 2012 | 95 | 1825 | 2198 |
| 70 | 2044 | 99 | 1850 | 2238 |
| 71 | 2077 | 103 | 1876 | 2279 |
| 72 | 2111 | 107 | 1901 | 2321 |
| 73 | 2145 | 111 | 1927 | 2363 |
| 74 | 2180 | 116 | 1952 | 2407 |
| 75 | 2215 | 120 | 1978 | 2451 |
| 76 | 2250 | 125 | 2005 | 2496 |
| 77 | 2286 | 130 | 2031 | 2542 |
| 78 | 2323 | 135 | 2058 | 2588 |
| 79 | 2360 | 141 | 2085 | 2636 |
| 80 | 2398 | 146 | 2112 | 2685 |

Table s9: Predicted costs (€) for the overweight estimated as the average effects at representative values of age for females.

| **Age** | Overweight | **SE** | Lower 95% bound | Upper 95% bond |
| --- | --- | --- | --- | --- |
| 20 | 989 | 66 | 859 | 1119 |
| 21 | 1006 | 66 | 877 | 1136 |
| 22 | 1024 | 66 | 895 | 1152 |
| 23 | 1041 | 65 | 913 | 1169 |
| 24 | 1059 | 65 | 932 | 1186 |
| 25 | 1077 | 65 | 950 | 1204 |
| 26 | 1095 | 64 | 969 | 1221 |
| 27 | 1114 | 64 | 989 | 1239 |
| 28 | 1133 | 63 | 1009 | 1257 |
| 29 | 1152 | 63 | 1028 | 1275 |
| 30 | 1171 | 63 | 1049 | 1294 |
| 31 | 1191 | 62 | 1069 | 1313 |
| 32 | 1211 | 62 | 1090 | 1332 |
| 33 | 1231 | 61 | 1111 | 1352 |
| 34 | 1252 | 61 | 1133 | 1371 |
| 35 | 1273 | 60 | 1155 | 1391 |
| 36 | 1294 | 60 | 1177 | 1412 |
| 37 | 1316 | 60 | 1199 | 1433 |
| 38 | 1338 | 59 | 1222 | 1454 |
| 39 | 1360 | 59 | 1245 | 1475 |
| 40 | 1383 | 59 | 1268 | 1497 |
| 41 | 1405 | 58 | 1291 | 1519 |
| 42 | 1429 | 58 | 1315 | 1542 |
| 43 | 1452 | 58 | 1339 | 1565 |
| 44 | 1476 | 58 | 1363 | 1589 |
| 45 | 1500 | 58 | 1388 | 1613 |
| 46 | 1525 | 58 | 1412 | 1638 |
| 47 | 1550 | 58 | 1437 | 1663 |
| 48 | 1575 | 58 | 1462 | 1689 |
| 49 | 1601 | 58 | 1487 | 1715 |
| 50 | 1627 | 58 | 1513 | 1742 |
| 51 | 1654 | 59 | 1538 | 1769 |
| 52 | 1681 | 60 | 1564 | 1797 |
| 53 | 1708 | 60 | 1590 | 1826 |
| 54 | 1736 | 61 | 1616 | 1856 |
| 55 | 1764 | 62 | 1642 | 1886 |
| 56 | 1792 | 63 | 1668 | 1917 |
| 57 | 1821 | 65 | 1694 | 1948 |
| 58 | 1851 | 66 | 1721 | 1981 |
| 59 | 1881 | 68 | 1747 | 2014 |
| 60 | 1911 | 70 | 1774 | 2048 |
| 61 | 1942 | 72 | 1800 | 2083 |
| 62 | 1973 | 74 | 1827 | 2118 |
| 63 | 2004 | 77 | 1854 | 2155 |
| 64 | 2036 | 79 | 1881 | 2192 |
| 65 | 2069 | 82 | 1908 | 2230 |
| 66 | 2102 | 85 | 1936 | 2269 |
| 67 | 2136 | 88 | 1963 | 2308 |
| 68 | 2170 | 91 | 1990 | 2349 |
| 69 | 2204 | 95 | 2018 | 2390 |
| 70 | 2239 | 99 | 2046 | 2433 |
| 71 | 2275 | 103 | 2074 | 2476 |
| 72 | 2311 | 107 | 2102 | 2520 |
| 73 | 2348 | 111 | 2130 | 2565 |
| 74 | 2385 | 115 | 2159 | 2611 |
| 75 | 2423 | 120 | 2188 | 2658 |
| 76 | 2461 | 125 | 2217 | 2705 |
| 77 | 2500 | 130 | 2246 | 2754 |
| 78 | 2539 | 135 | 2275 | 2804 |
| 79 | 2579 | 140 | 2305 | 2854 |
| 80 | 2620 | 146 | 2334 | 2906 |

Table s10: Predicted costs (€) for the obese grade 1 estimated as the average effects at representative values of age for females.

| Age | Obese grade 1 | **SE** | Lower 95% bound | Upper 95% bond |
| --- | --- | --- | --- | --- |
| 20 | 1267 | 96 | 1078 | 1456 |
| 21 | 1288 | 96 | 1099 | 1477 |
| 22 | 1310 | 96 | 1121 | 1499 |
| 23 | 1332 | 96 | 1143 | 1521 |
| 24 | 1355 | 97 | 1165 | 1544 |
| 25 | 1377 | 97 | 1188 | 1567 |
| 26 | 1400 | 97 | 1211 | 1590 |
| 27 | 1424 | 97 | 1234 | 1613 |
| 28 | 1448 | 97 | 1258 | 1637 |
| 29 | 1472 | 97 | 1282 | 1661 |
| 30 | 1496 | 97 | 1306 | 1686 |
| 31 | 1521 | 97 | 1331 | 1711 |
| 32 | 1546 | 97 | 1356 | 1737 |
| 33 | 1572 | 97 | 1381 | 1762 |
| 34 | 1598 | 97 | 1407 | 1789 |
| 35 | 1624 | 98 | 1433 | 1815 |
| 36 | 1651 | 98 | 1459 | 1843 |
| 37 | 1678 | 98 | 1486 | 1870 |
| 38 | 1706 | 98 | 1513 | 1899 |
| 39 | 1734 | 99 | 1540 | 1927 |
| 40 | 1762 | 99 | 1567 | 1956 |
| 41 | 1791 | 100 | 1595 | 1986 |
| 42 | 1820 | 100 | 1623 | 2017 |
| 43 | 1850 | 101 | 1652 | 2047 |
| 44 | 1880 | 102 | 1681 | 2079 |
| 45 | 1910 | 102 | 1710 | 2111 |
| 46 | 1941 | 103 | 1739 | 2144 |
| 47 | 1973 | 104 | 1768 | 2177 |
| 48 | 2005 | 105 | 1798 | 2211 |
| 49 | 2037 | 106 | 1828 | 2246 |
| 50 | 2070 | 108 | 1859 | 2281 |
| 51 | 2103 | 109 | 1889 | 2317 |
| 52 | 2137 | 111 | 1920 | 2354 |
| 53 | 2171 | 112 | 1951 | 2392 |
| 54 | 2206 | 114 | 1982 | 2430 |
| 55 | 2242 | 116 | 2014 | 2470 |
| 56 | 2278 | 118 | 2046 | 2510 |
| 57 | 2314 | 121 | 2078 | 2551 |
| 58 | 2351 | 123 | 2110 | 2592 |
| 59 | 2389 | 126 | 2142 | 2635 |
| 60 | 2427 | 128 | 2175 | 2679 |
| 61 | 2465 | 131 | 2208 | 2723 |
| 62 | 2505 | 135 | 2241 | 2768 |
| 63 | 2544 | 138 | 2274 | 2815 |
| 64 | 2585 | 141 | 2308 | 2862 |
| 65 | 2626 | 145 | 2341 | 2910 |
| 66 | 2667 | 149 | 2375 | 2960 |
| 67 | 2710 | 153 | 2409 | 3010 |
| 68 | 2752 | 157 | 2444 | 3061 |
| 69 | 2796 | 162 | 2478 | 3113 |
| 70 | 2840 | 167 | 2513 | 3167 |
| 71 | 2885 | 172 | 2548 | 3221 |
| 72 | 2930 | 177 | 2584 | 3277 |
| 73 | 2976 | 182 | 2619 | 3333 |
| 74 | 3023 | 188 | 2655 | 3391 |
| 75 | 3071 | 194 | 2691 | 3450 |
| 76 | 3119 | 200 | 2728 | 3510 |
| 77 | 3168 | 206 | 2764 | 3571 |
| 78 | 3217 | 212 | 2801 | 3634 |
| 79 | 3268 | 219 | 2838 | 3697 |
| 80 | 3319 | 226 | 2876 | 3762 |

Table s11: Predicted costs (€) for the obese grade 2 estimated as the average effects at representative values of age for females.

| Age | Obese grade 2 | **SE** | Lower 95% bound | Upper 95% bond |
| --- | --- | --- | --- | --- |
| 20 | 1331 | 131 | 1075 | 1587 |
| 21 | 1352 | 131 | 1095 | 1610 |
| 22 | 1374 | 132 | 1115 | 1633 |
| 23 | 1396 | 133 | 1135 | 1657 |
| 24 | 1419 | 134 | 1156 | 1682 |
| 25 | 1442 | 135 | 1177 | 1706 |
| 26 | 1465 | 136 | 1198 | 1731 |
| 27 | 1488 | 137 | 1220 | 1757 |
| 28 | 1512 | 138 | 1242 | 1783 |
| 29 | 1536 | 139 | 1264 | 1809 |
| 30 | 1561 | 140 | 1286 | 1836 |
| 31 | 1586 | 141 | 1309 | 1863 |
| 32 | 1611 | 143 | 1332 | 1891 |
| 33 | 1637 | 144 | 1355 | 1919 |
| 34 | 1663 | 145 | 1379 | 1947 |
| 35 | 1689 | 146 | 1402 | 1976 |
| 36 | 1716 | 148 | 1427 | 2006 |
| 37 | 1743 | 149 | 1451 | 2036 |
| 38 | 1771 | 151 | 1476 | 2066 |
| 39 | 1799 | 152 | 1501 | 2097 |
| 40 | 1828 | 154 | 1526 | 2129 |
| 41 | 1856 | 156 | 1552 | 2161 |
| 42 | 1886 | 157 | 1577 | 2194 |
| 43 | 1915 | 159 | 1604 | 2227 |
| 44 | 1946 | 161 | 1630 | 2261 |
| 45 | 1976 | 163 | 1657 | 2296 |
| 46 | 2007 | 165 | 1684 | 2331 |
| 47 | 2039 | 167 | 1711 | 2367 |
| 48 | 2071 | 170 | 1739 | 2403 |
| 49 | 2103 | 172 | 1767 | 2440 |
| 50 | 2136 | 174 | 1795 | 2478 |
| 51 | 2170 | 177 | 1823 | 2516 |
| 52 | 2204 | 180 | 1852 | 2556 |
| 53 | 2238 | 182 | 1881 | 2596 |
| 54 | 2273 | 185 | 1910 | 2636 |
| 55 | 2309 | 188 | 1940 | 2678 |
| 56 | 2345 | 191 | 1970 | 2720 |
| 57 | 2381 | 195 | 2000 | 2763 |
| 58 | 2418 | 198 | 2030 | 2807 |
| 59 | 2456 | 202 | 2061 | 2851 |
| 60 | 2494 | 205 | 2092 | 2897 |
| 61 | 2533 | 209 | 2123 | 2943 |
| 62 | 2572 | 213 | 2154 | 2990 |
| 63 | 2612 | 217 | 2186 | 3038 |
| 64 | 2653 | 222 | 2218 | 3087 |
| 65 | 2694 | 226 | 2251 | 3137 |
| 66 | 2736 | 231 | 2283 | 3188 |
| 67 | 2778 | 236 | 2316 | 3240 |
| 68 | 2821 | 241 | 2349 | 3292 |
| 69 | 2865 | 246 | 2383 | 3346 |
| 70 | 2909 | 251 | 2417 | 3401 |
| 71 | 2954 | 257 | 2451 | 3457 |
| 72 | 2999 | 262 | 2485 | 3514 |
| 73 | 3046 | 268 | 2520 | 3571 |
| 74 | 3093 | 274 | 2555 | 3630 |
| 75 | 3140 | 281 | 2590 | 3691 |
| 76 | 3189 | 287 | 2625 | 3752 |
| 77 | 3238 | 294 | 2661 | 3814 |
| 78 | 3287 | 301 | 2697 | 3878 |
| 79 | 3338 | 308 | 2734 | 3942 |
| 80 | 3389 | 316 | 2771 | 4008 |

Table s12: Predicted costs (€) for the normal weight estimated as the average effects at representative values of age for males.

| Age | Normal weight | **SE** | Lower 95% bound | Upper 95% bond |
| --- | --- | --- | --- | --- |
| 20 | 355 | 30 | 296 | 415 |
| 21 | 373 | 31 | 311 | 434 |
| 22 | 390 | 32 | 327 | 453 |
| 23 | 409 | 33 | 344 | 473 |
| 24 | 428 | 34 | 362 | 494 |
| 25 | 448 | 35 | 380 | 516 |
| 26 | 469 | 35 | 400 | 538 |
| 27 | 490 | 36 | 419 | 561 |
| 28 | 513 | 37 | 440 | 585 |
| 29 | 536 | 38 | 461 | 610 |
| 30 | 560 | 39 | 484 | 636 |
| 31 | 585 | 40 | 507 | 662 |
| 32 | 610 | 41 | 531 | 690 |
| 33 | 637 | 41 | 556 | 718 |
| 34 | 664 | 42 | 581 | 747 |
| 35 | 693 | 43 | 608 | 778 |
| 36 | 722 | 44 | 636 | 809 |
| 37 | 753 | 45 | 664 | 842 |
| 38 | 785 | 46 | 694 | 875 |
| 39 | 817 | 47 | 724 | 910 |
| 40 | 851 | 49 | 756 | 946 |
| 41 | 886 | 50 | 788 | 983 |
| 42 | 922 | 51 | 822 | 1022 |
| 43 | 959 | 52 | 857 | 1061 |
| 44 | 997 | 54 | 892 | 1103 |
| 45 | 1037 | 55 | 929 | 1145 |
| 46 | 1078 | 57 | 967 | 1189 |
| 47 | 1120 | 58 | 1006 | 1235 |
| 48 | 1164 | 60 | 1046 | 1282 |
| 49 | 1209 | 62 | 1088 | 1331 |
| 50 | 1256 | 64 | 1130 | 1381 |
| 51 | 1303 | 66 | 1174 | 1433 |
| 52 | 1353 | 69 | 1219 | 1487 |
| 53 | 1404 | 71 | 1265 | 1543 |
| 54 | 1457 | 74 | 1312 | 1601 |
| 55 | 1511 | 77 | 1360 | 1661 |
| 56 | 1567 | 80 | 1410 | 1723 |
| 57 | 1624 | 83 | 1461 | 1788 |
| 58 | 1684 | 87 | 1513 | 1854 |
| 59 | 1745 | 91 | 1567 | 1923 |
| 60 | 1808 | 95 | 1622 | 1995 |
| 61 | 1873 | 100 | 1678 | 2068 |
| 62 | 1940 | 104 | 1736 | 2145 |
| 63 | 2010 | 109 | 1795 | 2224 |
| 64 | 2081 | 115 | 1856 | 2306 |
| 65 | 2154 | 121 | 1918 | 2391 |
| 66 | 2230 | 127 | 1981 | 2479 |
| 67 | 2308 | 134 | 2046 | 2570 |
| 68 | 2388 | 141 | 2113 | 2664 |
| 69 | 2471 | 148 | 2181 | 2761 |
| 70 | 2556 | 156 | 2251 | 2862 |
| 71 | 2644 | 164 | 2322 | 2966 |
| 72 | 2735 | 173 | 2395 | 3074 |
| 73 | 2828 | 182 | 2470 | 3186 |
| 74 | 2924 | 192 | 2547 | 3301 |
| 75 | 3023 | 203 | 2626 | 3420 |
| 76 | 3125 | 214 | 2706 | 3544 |
| 77 | 3230 | 225 | 2789 | 3671 |
| 78 | 3338 | 237 | 2873 | 3804 |
| 79 | 3450 | 250 | 2960 | 3940 |
| 80 | 3565 | 264 | 3048 | 4081 |

Table s13: Predicted costs (€) for the overweight estimated as the average effects at representative values of age for males.

| **Age** | Overweight | **SE** | Lower 95% bound | Upper 95% bond |
| --- | --- | --- | --- | --- |
| 20 | 344 | 27 | 290 | 398 |
| 21 | 360 | 28 | 306 | 415 |
| 22 | 377 | 28 | 321 | 433 |
| 23 | 395 | 29 | 338 | 452 |
| 24 | 413 | 30 | 355 | 471 |
| 25 | 432 | 30 | 373 | 491 |
| 26 | 451 | 31 | 391 | 511 |
| 27 | 471 | 31 | 410 | 532 |
| 28 | 492 | 32 | 430 | 554 |
| 29 | 514 | 32 | 451 | 577 |
| 30 | 537 | 33 | 473 | 601 |
| 31 | 560 | 33 | 495 | 625 |
| 32 | 584 | 33 | 518 | 650 |
| 33 | 609 | 34 | 543 | 676 |
| 34 | 635 | 34 | 568 | 702 |
| 35 | 662 | 35 | 594 | 730 |
| 36 | 689 | 35 | 620 | 758 |
| 37 | 718 | 35 | 648 | 787 |
| 38 | 747 | 36 | 677 | 818 |
| 39 | 778 | 36 | 707 | 849 |
| 40 | 809 | 37 | 737 | 881 |
| 41 | 842 | 37 | 769 | 914 |
| 42 | 875 | 37 | 802 | 949 |
| 43 | 910 | 38 | 836 | 984 |
| 44 | 946 | 38 | 871 | 1021 |
| 45 | 983 | 39 | 907 | 1059 |
| 46 | 1021 | 39 | 944 | 1098 |
| 47 | 1060 | 40 | 982 | 1139 |
| 48 | 1101 | 41 | 1021 | 1181 |
| 49 | 1143 | 42 | 1061 | 1224 |
| 50 | 1186 | 43 | 1102 | 1269 |
| 51 | 1230 | 44 | 1145 | 1316 |
| 52 | 1276 | 45 | 1188 | 1364 |
| 53 | 1324 | 46 | 1233 | 1414 |
| 54 | 1372 | 48 | 1279 | 1466 |
| 55 | 1423 | 50 | 1326 | 1520 |
| 56 | 1475 | 52 | 1374 | 1576 |
| 57 | 1528 | 54 | 1423 | 1633 |
| 58 | 1583 | 56 | 1473 | 1694 |
| 59 | 1640 | 59 | 1524 | 1756 |
| 60 | 1699 | 62 | 1577 | 1820 |
| 61 | 1759 | 65 | 1631 | 1887 |
| 62 | 1821 | 69 | 1686 | 1957 |
| 63 | 1885 | 73 | 1742 | 2029 |
| 64 | 1951 | 77 | 1799 | 2103 |
| 65 | 2019 | 82 | 1858 | 2181 |
| 66 | 2090 | 87 | 1918 | 2261 |
| 67 | 2162 | 93 | 1980 | 2344 |
| 68 | 2236 | 99 | 2043 | 2429 |
| 69 | 2313 | 105 | 2107 | 2518 |
| 70 | 2392 | 112 | 2173 | 2611 |
| 71 | 2473 | 119 | 2241 | 2706 |
| 72 | 2557 | 126 | 2310 | 2805 |
| 73 | 2644 | 134 | 2380 | 2907 |
| 74 | 2733 | 143 | 2453 | 3012 |
| 75 | 2824 | 152 | 2527 | 3122 |
| 76 | 2919 | 161 | 2603 | 3235 |
| 77 | 3016 | 171 | 2680 | 3352 |
| 78 | 3116 | 182 | 2760 | 3473 |
| 79 | 3219 | 193 | 2841 | 3598 |
| 80 | 3326 | 205 | 2924 | 3727 |

Table s14: Predicted costs (€) for the obese grade 1 estimated as the average effects at representative values of age for males.

| Age | Obese grade 1 | **SE** | Lower 95% bound | Upper 95% bond |
| --- | --- | --- | --- | --- |
| 20 | 447 | 40 | 368 | 527 |
| 21 | 467 | 41 | 386 | 548 |
| 22 | 487 | 42 | 404 | 570 |
| 23 | 508 | 43 | 423 | 593 |
| 24 | 530 | 44 | 443 | 617 |
| 25 | 552 | 45 | 463 | 641 |
| 26 | 575 | 46 | 485 | 666 |
| 27 | 599 | 47 | 507 | 692 |
| 28 | 624 | 48 | 529 | 719 |
| 29 | 650 | 49 | 553 | 746 |
| 30 | 676 | 50 | 577 | 775 |
| 31 | 704 | 51 | 603 | 804 |
| 32 | 732 | 53 | 629 | 835 |
| 33 | 761 | 54 | 656 | 866 |
| 34 | 791 | 55 | 684 | 898 |
| 35 | 822 | 56 | 712 | 932 |
| 36 | 854 | 57 | 742 | 966 |
| 37 | 887 | 58 | 773 | 1001 |
| 38 | 921 | 59 | 805 | 1038 |
| 39 | 956 | 61 | 837 | 1076 |
| 40 | 993 | 62 | 871 | 1114 |
| 41 | 1030 | 63 | 906 | 1155 |
| 42 | 1069 | 65 | 942 | 1196 |
| 43 | 1109 | 66 | 978 | 1239 |
| 44 | 1150 | 68 | 1016 | 1283 |
| 45 | 1192 | 70 | 1055 | 1329 |
| 46 | 1236 | 71 | 1096 | 1376 |
| 47 | 1281 | 73 | 1137 | 1424 |
| 48 | 1327 | 75 | 1179 | 1475 |
| 49 | 1375 | 77 | 1223 | 1527 |
| 50 | 1424 | 80 | 1268 | 1580 |
| 51 | 1475 | 82 | 1314 | 1636 |
| 52 | 1527 | 85 | 1361 | 1693 |
| 53 | 1581 | 87 | 1410 | 1752 |
| 54 | 1637 | 90 | 1460 | 1814 |
| 55 | 1694 | 94 | 1511 | 1877 |
| 56 | 1753 | 97 | 1563 | 1943 |
| 57 | 1814 | 100 | 1617 | 2011 |
| 58 | 1876 | 104 | 1672 | 2081 |
| 59 | 1941 | 108 | 1728 | 2154 |
| 60 | 2007 | 113 | 1786 | 2229 |
| 61 | 2076 | 118 | 1845 | 2306 |
| 62 | 2146 | 123 | 1906 | 2387 |
| 63 | 2219 | 128 | 1968 | 2470 |
| 64 | 2294 | 134 | 2032 | 2556 |
| 65 | 2371 | 140 | 2097 | 2645 |
| 66 | 2451 | 146 | 2164 | 2737 |
| 67 | 2533 | 153 | 2232 | 2833 |
| 68 | 2617 | 160 | 2302 | 2931 |
| 69 | 2704 | 168 | 2374 | 3033 |
| 70 | 2793 | 176 | 2448 | 3139 |
| 71 | 2885 | 185 | 2523 | 3248 |
| 72 | 2980 | 194 | 2600 | 3361 |
| 73 | 3078 | 204 | 2679 | 3478 |
| 74 | 3179 | 214 | 2759 | 3599 |
| 75 | 3283 | 225 | 2842 | 3724 |
| 76 | 3390 | 236 | 2927 | 3853 |
| 77 | 3500 | 248 | 3014 | 3987 |
| 78 | 3614 | 261 | 3102 | 4125 |
| 79 | 3731 | 274 | 3193 | 4268 |
| 80 | 3851 | 288 | 3287 | 4416 |

Table s15: Predicted costs (€) for the obese grade 2 estimated as the average effects at representative values of age for males.

| Age | Obese grade 2 | **SE** | Lower 95% bound | Upper 95% bond |
| --- | --- | --- | --- | --- |
| 20 | 842 | 117 | 613 | 1071 |
| 21 | 875 | 120 | 640 | 1111 |
| 22 | 910 | 124 | 667 | 1153 |
| 23 | 946 | 128 | 695 | 1197 |
| 24 | 983 | 132 | 724 | 1241 |
| 25 | 1021 | 136 | 754 | 1287 |
| 26 | 1060 | 140 | 785 | 1335 |
| 27 | 1100 | 145 | 817 | 1384 |
| 28 | 1142 | 149 | 850 | 1434 |
| 29 | 1185 | 154 | 884 | 1487 |
| 30 | 1230 | 158 | 919 | 1540 |
| 31 | 1276 | 163 | 955 | 1596 |
| 32 | 1323 | 168 | 993 | 1653 |
| 33 | 1372 | 174 | 1031 | 1712 |
| 34 | 1422 | 179 | 1071 | 1773 |
| 35 | 1474 | 185 | 1112 | 1836 |
| 36 | 1527 | 190 | 1154 | 1900 |
| 37 | 1582 | 196 | 1197 | 1967 |
| 38 | 1639 | 203 | 1242 | 2036 |
| 39 | 1697 | 209 | 1288 | 2107 |
| 40 | 1757 | 216 | 1335 | 2180 |
| 41 | 1819 | 222 | 1384 | 2255 |
| 42 | 1883 | 229 | 1434 | 2333 |
| 43 | 1949 | 237 | 1485 | 2413 |
| 44 | 2017 | 244 | 1538 | 2496 |
| 45 | 2087 | 252 | 1593 | 2581 |
| 46 | 2159 | 260 | 1649 | 2670 |
| 47 | 2233 | 269 | 1707 | 2760 |
| 48 | 2310 | 278 | 1766 | 2854 |
| 49 | 2389 | 287 | 1827 | 2951 |
| 50 | 2470 | 296 | 1889 | 3051 |
| 51 | 2554 | 306 | 1954 | 3154 |
| 52 | 2640 | 316 | 2020 | 3260 |
| 53 | 2729 | 327 | 2088 | 3369 |
| 54 | 2820 | 338 | 2158 | 3482 |
| 55 | 2914 | 349 | 2230 | 3599 |
| 56 | 3011 | 361 | 2303 | 3720 |
| 57 | 3111 | 374 | 2379 | 3844 |
| 58 | 3214 | 387 | 2457 | 3972 |
| 59 | 3321 | 400 | 2536 | 4105 |
| 60 | 3430 | 414 | 2618 | 4241 |
| 61 | 3543 | 429 | 2703 | 4383 |
| 62 | 3659 | 444 | 2789 | 4528 |
| 63 | 3778 | 459 | 2878 | 4679 |
| 64 | 3901 | 476 | 2969 | 4834 |
| 65 | 4028 | 493 | 3062 | 4994 |
| 66 | 4159 | 511 | 3158 | 5160 |
| 67 | 4293 | 529 | 3256 | 5330 |
| 68 | 4432 | 548 | 3357 | 5507 |
| 69 | 4575 | 568 | 3461 | 5689 |
| 70 | 4722 | 589 | 3567 | 5877 |
| 71 | 4874 | 611 | 3676 | 6071 |
| 72 | 5030 | 634 | 3788 | 6272 |
| 73 | 5191 | 657 | 3903 | 6479 |
| 74 | 5357 | 682 | 4021 | 6693 |
| 75 | 5528 | 707 | 4142 | 6914 |
| 76 | 5704 | 734 | 4265 | 7142 |
| 77 | 5885 | 761 | 4392 | 7377 |
| 78 | 6072 | 790 | 4523 | 7621 |
| 79 | 6264 | 820 | 4656 | 7872 |
| 80 | 6463 | 852 | 4793 | 8132 |

# Supplementary Appendix 5 - Transition probabilities

Table s16: Transition probabilities (for females) between the health states.

|  |  | **N to OW** | **OW to OB1** | **OB1 to OB2** | **OW to N** | **OB1 to OW** | **OB 2 to OB 1** |
| --- | --- | --- | --- | --- | --- | --- | --- |
| **2 -** | **3 Years** | 0,49 % | 1,42 % | 1,94 % | 4,21 % | 8,18 % | 5,03 % |
| **3 -** | **4 Years** | 1,70 % | 2,77 % | 3,19 % | 12,26 % | 19,50 % | 18,57 % |
| **4 -** | **5 Years** | 2,46 % | 3,12 % | 3,35 % | 14,89 % | 21,60 % | 23,67 % |
| **5 -** | **6 Years** | 2,88 % | 3,20 % | 3,32 % | 15,45 % | 21,39 % | 24,84 % |
| **6 -** | **7 Years** | 3,11 % | 3,18 % | 3,22 % | 15,31 % | 20,60 % | 24,71 % |
| **7 -** | **8 Years** | 3,24 % | 3,12 % | 3,11 % | 14,91 % | 19,68 % | 24,09 % |
| **8 -** | **9 Years** | 3,31 % | 3,05 % | 3,00 % | 14,42 % | 18,76 % | 23,31 % |
| **9 -** | **10 Years** | 3,33 % | 2,97 % | 2,90 % | 13,91 % | 17,90 % | 22,49 % |
| **10 -** | **11 Years** | 3,33 % | 2,89 % | 2,80 % | 13,42 % | 17,11 % | 21,69 % |
| **11 -** | **12 Years** | 3,31 % | 2,81 % | 2,70 % | 12,94 % | 16,39 % | 20,91 % |
| **12 -** | **13 Years** | 0,18 % | 0,54 % | 2,18 % | 17,10 % | 22,50 % | 33,61 % |
| **13 -** | **14 Years** | 0,96 % | 1,31 % | 2,45 % | 13,02 % | 15,21 % | 25,08 % |
| **14 -** | **15 Years** | 1,27 % | 1,94 % | 2,76 % | 9,86 % | 10,13 % | 18,43 % |
| **15 -** | **16 Years** | 1,70 % | 2,51 % | 3,10 % | 7,43 % | 6,68 % | 13,38 % |
| **16 -** | **17 Years** | 2,10 % | 3,04 % | 3,48 % | 5,58 % | 4,37 % | 9,63 % |
| **17 -** | **18 Years** | 2,49 % | 3,54 % | 3,91 % | 4,18 % | 2,85 % | 6,89 % |
| **18 -** | **19 Years** | 2,86 % | 4,02 % | 4,39 % | 3,13 % | 1,86 % | 4,91 % |
| **19 -** | **20 Years** | 3,23 % | 4,49 % | 4,92 % | 2,34 % | 1,21 % | 3,49 % |
| **20 -** | **21 Years** | 0,28 % | 0,96 % | 4,61 % | 0,15 % | 0,24 % | 0,81 % |
| **21 -** | **22 Years** | 0,52 % | 1,31 % | 4,52 % | 0,16 % | 0,24 % | 0,82 % |
| **22 -** | **23 Years** | 0,78 % | 1,59 % | 4,44 % | 0,16 % | 0,25 % | 0,84 % |
| **23 -** | **24 Years** | 1,05 % | 1,83 % | 4,35 % | 0,17 % | 0,26 % | 0,86 % |
| **24 -** | **25 Years** | 1,32 % | 2,03 % | 4,27 % | 0,18 % | 0,27 % | 0,88 % |
| **25 -** | **26 Years** | 1,59 % | 2,20 % | 4,19 % | 0,18 % | 0,28 % | 0,90 % |
| **26 -** | **27 Years** | 1,85 % | 2,35 % | 4,10 % | 0,19 % | 0,28 % | 0,92 % |
| **27 -** | **28 Years** | 2,11 % | 2,48 % | 4,03 % | 0,20 % | 0,29 % | 0,94 % |
| **28 -** | **29 Years** | 2,36 % | 2,59 % | 3,95 % | 0,20 % | 0,30 % | 0,96 % |
| **29 -** | **30 Years** | 2,60 % | 2,69 % | 3,87 % | 0,21 % | 0,31 % | 0,98 % |
| **30 -** | **31 Years** | 2,82 % | 2,77 % | 3,80 % | 0,22 % | 0,32 % | 1,00 % |
| **31 -** | **32 Years** | 3,02 % | 2,83 % | 3,72 % | 0,23 % | 0,33 % | 1,03 % |
| **32 -** | **33 Years** | 3,21 % | 2,89 % | 3,65 % | 0,24 % | 0,34 % | 1,05 % |
| **33 -** | **34 Years** | 3,39 % | 2,93 % | 3,58 % | 0,25 % | 0,35 % | 1,07 % |
| **34 -** | **35 Years** | 3,54 % | 2,96 % | 3,51 % | 0,26 % | 0,36 % | 1,10 % |
| **35 -** | **36 Years** | 3,68 % | 2,99 % | 3,44 % | 0,27 % | 0,37 % | 1,12 % |
| **36 -** | **37 Years** | 3,80 % | 3,01 % | 3,38 % | 0,28 % | 0,38 % | 1,15 % |
| **37 -** | **38 Years** | 3,91 % | 3,02 % | 3,31 % | 0,29 % | 0,39 % | 1,17 % |
| **38 -** | **39 Years** | 4,00 % | 3,03 % | 3,25 % | 0,30 % | 0,41 % | 1,20 % |
| **39 -** | **40 Years** | 4,08 % | 3,03 % | 3,18 % | 0,31 % | 0,42 % | 1,22 % |
| **40 -** | **41 Years** | 4,14 % | 3,03 % | 3,12 % | 0,33 % | 0,43 % | 1,25 % |
| **41 -** | **42 Years** | 4,19 % | 3,02 % | 3,06 % | 0,34 % | 0,44 % | 1,28 % |
| **42 -** | **43 Years** | 4,23 % | 3,01 % | 3,00 % | 0,35 % | 0,46 % | 1,31 % |
| **43 -** | **44 Years** | 4,26 % | 3,00 % | 2,95 % | 0,37 % | 0,47 % | 1,34 % |
| **44 -** | **45 Years** | 4,28 % | 2,98 % | 2,89 % | 0,38 % | 0,48 % | 1,37 % |
| **45 -** | **46 Years** | 4,29 % | 2,97 % | 2,83 % | 0,40 % | 0,50 % | 1,40 % |
| **46 -** | **47 Years** | 4,30 % | 2,95 % | 2,78 % | 0,41 % | 0,51 % | 1,43 % |
| **47 -** | **48 Years** | 4,30 % | 2,92 % | 2,72 % | 0,43 % | 0,53 % | 1,46 % |
| **48 -** | **49 Years** | 4,29 % | 2,90 % | 2,67 % | 0,44 % | 0,55 % | 1,49 % |
| **49 -** | **50 Years** | 4,27 % | 2,88 % | 2,62 % | 0,46 % | 0,56 % | 1,52 % |
| **50 -** | **51 Years** | 4,26 % | 2,85 % | 2,57 % | 0,48 % | 0,58 % | 1,56 % |
| **51 -** | **52 Years** | 4,23 % | 2,83 % | 2,52 % | 0,50 % | 0,60 % | 1,59 % |
| **52 -** | **53 Years** | 4,21 % | 2,80 % | 2,47 % | 0,52 % | 0,61 % | 1,63 % |
| **53 -** | **54 Years** | 4,18 % | 2,77 % | 2,42 % | 0,54 % | 0,63 % | 1,66 % |
| **54 -** | **55 Years** | 4,15 % | 2,75 % | 2,37 % | 0,56 % | 0,65 % | 1,70 % |
| **55 -** | **56 Years** | 4,11 % | 2,72 % | 2,33 % | 0,58 % | 0,67 % | 1,74 % |
| **56 -** | **57 Years** | 4,08 % | 2,69 % | 2,28 % | 0,61 % | 0,69 % | 1,78 % |
| **57 -** | **58 Years** | 4,04 % | 2,66 % | 2,24 % | 0,63 % | 0,71 % | 1,82 % |
| **58 -** | **59 Years** | 4,00 % | 2,63 % | 2,19 % | 0,65 % | 0,73 % | 1,86 % |
| **59 -** | **60 Years** | 3,96 % | 2,61 % | 2,15 % | 0,68 % | 0,76 % | 1,90 % |
| **60 -** | **61 Years** | 3,92 % | 2,58 % | 2,11 % | 0,71 % | 0,78 % | 1,94 % |
| **61 -** | **62 Years** | 3,88 % | 2,55 % | 2,07 % | 0,74 % | 0,80 % | 1,98 % |
| **62 -** | **63 Years** | 3,84 % | 2,52 % | 2,03 % | 0,76 % | 0,83 % | 2,03 % |
| **63 -** | **64 Years** | 3,79 % | 2,49 % | 1,99 % | 0,79 % | 0,85 % | 2,07 % |
| **64 -** | **65 Years** | 3,75 % | 2,47 % | 1,95 % | 0,83 % | 0,88 % | 2,12 % |
| **65 -** | **66 Years** | 3,71 % | 2,44 % | 1,91 % | 0,86 % | 0,90 % | 2,17 % |
| **66 -** | **67 Years** | 3,67 % | 2,41 % | 1,87 % | 0,89 % | 0,93 % | 2,21 % |
| **67 -** | **68 Years** | 3,62 % | 2,39 % | 1,84 % | 0,93 % | 0,96 % | 2,26 % |
| **68 -** | **69 Years** | 3,58 % | 2,36 % | 1,80 % | 0,96 % | 0,99 % | 2,31 % |
| **69 -** | **70 Years** | 3,54 % | 2,33 % | 1,77 % | 1,00 % | 1,01 % | 2,36 % |
| **70 -** | **71 Years** | 3,50 % | 2,31 % | 1,73 % | 1,04 % | 1,05 % | 2,42 % |
| **71 -** | **72 Years** | 3,46 % | 2,28 % | 1,70 % | 1,08 % | 1,08 % | 2,47 % |
| **72 -** | **73 Years** | 3,42 % | 2,26 % | 1,67 % | 1,13 % | 1,11 % | 2,52 % |
| **73 -** | **74 Years** | 3,38 % | 2,23 % | 1,63 % | 1,17 % | 1,14 % | 2,58 % |
| **74 -** | **75 Years** | 3,34 % | 2,21 % | 1,60 % | 1,22 % | 1,18 % | 2,64 % |
| **75 -** | **76 Years** | 3,30 % | 2,19 % | 1,57 % | 1,26 % | 1,21 % | 2,70 % |
| **76 -** | **77 Years** | 3,26 % | 2,16 % | 1,54 % | 1,31 % | 1,25 % | 2,75 % |
| **77 -** | **78 Years** | 3,22 % | 2,14 % | 1,51 % | 1,37 % | 1,29 % | 2,82 % |
| **78 -** | **79 Years** | 3,18 % | 2,12 % | 1,48 % | 1,42 % | 1,32 % | 2,88 % |
| **79 -** | **80 Years** | 3,15 % | 2,09 % | 1,45 % | 1,47 % | 1,36 % | 2,94 % |
| **80 -** | **81 Years** | 3,11 % | 2,07 % | 1,42 % | 1,53 % | 1,40 % | 3,01 % |
| **81 -** | **82 Years** | 3,07 % | 2,05 % | 1,39 % | 1,59 % | 1,45 % | 3,07 % |
| **82 -** | **83 Years** | 3,04 % | 2,03 % | 1,37 % | 1,66 % | 1,49 % | 3,14 % |
| **83 -** | **84 Years** | 3,00 % | 2,01 % | 1,34 % | 1,72 % | 1,53 % | 3,21 % |
| **84 -** | **85 Years** | 2,97 % | 1,99 % | 1,31 % | 1,79 % | 1,58 % | 3,28 % |
| **85 -** | **86 Years** | 2,93 % | 1,97 % | 1,29 % | 1,86 % | 1,63 % | 3,35 % |
| **86 -** | **87 Years** | 2,90 % | 1,95 % | 1,26 % | 1,93 % | 1,68 % | 3,43 % |
| **87 -** | **88 Years** | 2,87 % | 1,93 % | 1,24 % | 2,01 % | 1,73 % | 3,50 % |
| **88 -** | **89 Years** | 2,84 % | 1,91 % | 1,21 % | 2,09 % | 1,78 % | 3,58 % |
| **89 -** | **90 Years** | 2,81 % | 1,89 % | 1,19 % | 2,17 % | 1,83 % | 3,66 % |
| **90 -** | **91 Years** | 2,78 % | 1,87 % | 1,17 % | 2,25 % | 1,88 % | 3,74 % |
| **91 -** | **92 Years** | 2,74 % | 1,85 % | 1,14 % | 2,34 % | 1,94 % | 3,82 % |
| **92 -** | **93 Years** | 2,72 % | 1,83 % | 1,12 % | 2,43 % | 2,00 % | 3,90 % |
| **93 -** | **94 Years** | 2,69 % | 1,81 % | 1,10 % | 2,53 % | 2,06 % | 3,99 % |
| **94 -** | **95 Years** | 2,66 % | 1,80 % | 1,08 % | 2,63 % | 2,12 % | 4,08 % |
| **95 -** | **96 Years** | 2,63 % | 1,78 % | 1,06 % | 2,73 % | 2,18 % | 4,17 % |
| **96 -** | **97 Years** | 2,60 % | 1,76 % | 1,04 % | 2,84 % | 2,25 % | 4,26 % |
| **97 -** | **98 Years** | 2,57 % | 1,74 % | 1,02 % | 2,95 % | 2,32 % | 4,35 % |
| **98 -** | **99 Years** | 2,55 % | 1,73 % | 1,00 % | 3,06 % | 2,38 % | 4,44 % |
| **99 -** | **100 Years** | 2,52 % | 1,71 % | 0,98 % | 3,18 % | 2,46 % | 4,54 % |

*N = Normal weight, OW = Overweight, OB1 = obese grade 1, OB2 = obese grade 2*

Table s17: Transition probabilities (for males) between the health states.

|  |  | **N to OW** | **OW to OB1** | **OB1 to OB2** | **OW to N** | **OB1 to OW** | **OB2 to OB1** |
| --- | --- | --- | --- | --- | --- | --- | --- |
| **2 -** | **3 Years** | 0,31 % | 0,63 % | 1,69 % | 5,78 % | 7,83 % | 5,14 % |
| **3 -** | **4 Years** | 1,25 % | 1,84 % | 2,87 % | 15,82 % | 19,86 % | 16,80 % |
| **4 -** | **5 Years** | 1,94 % | 2,46 % | 3,07 % | 18,53 % | 22,40 % | 20,76 % |
| **5 -** | **6 Years** | 2,37 % | 2,77 % | 3,06 % | 18,84 % | 22,35 % | 21,57 % |
| **6 -** | **7 Years** | 2,64 % | 2,92 % | 2,99 % | 18,43 % | 21,61 % | 21,34 % |
| **7 -** | **8 Years** | 2,81 % | 2,99 % | 2,90 % | 17,80 % | 20,70 % | 20,75 % |
| **8 -** | **9 Years** | 2,91 % | 3,02 % | 2,81 % | 17,10 % | 19,77 % | 20,04 % |
| **9 -** | **10 Years** | 2,96 % | 3,01 % | 2,72 % | 16,42 % | 18,90 % | 19,31 % |
| **10 -** | **11 Years** | 2,99 % | 2,99 % | 2,63 % | 15,77 % | 18,08 % | 18,59 % |
| **11 -** | **12 Years** | 3,00 % | 2,96 % | 2,55 % | 15,16 % | 17,33 % | 17,91 % |
| **12 -** | **13 Years** | 0,10 % | 1,07 % | 4,69 % | 17,11 % | 12,91 % | 13,45 % |
| **13 -** | **14 Years** | 0,42 % | 2,05 % | 4,77 % | 12,24 % | 9,56 % | 11,61 % |
| **14 -** | **15 Years** | 0,85 % | 2,70 % | 4,85 % | 8,68 % | 7,04 % | 10,02 % |
| **15 -** | **16 Years** | 1,36 % | 3,23 % | 4,93 % | 6,12 % | 5,17 % | 8,63 % |
| **16 -** | **17 Years** | 1,93 % | 3,70 % | 5,02 % | 4,30 % | 3,78 % | 7,42 % |
| **17 -** | **18 Years** | 2,56 % | 4,12 % | 5,10 % | 3,01 % | 2,76 % | 6,38 % |
| **18 -** | **19 Years** | 3,23 % | 4,50 % | 5,19 % | 2,10 % | 2,01 % | 5,48 % |
| **19 -** | **20 Years** | 3,94 % | 4,85 % | 5,28 % | 1,47 % | 1,47 % | 4,70 % |
| **20 -** | **21 Years** | 1,04 % | 1,04 % | 2,30 % | 0,09 % | 0,17 % | 0,44 % |
| **21 -** | **22 Years** | 1,70 % | 1,24 % | 2,26 % | 0,10 % | 0,18 % | 0,46 % |
| **22 -** | **23 Years** | 2,32 % | 1,39 % | 2,23 % | 0,10 % | 0,19 % | 0,47 % |
| **23 -** | **24 Years** | 2,89 % | 1,50 % | 2,20 % | 0,11 % | 0,20 % | 0,49 % |
| **24 -** | **25 Years** | 3,41 % | 1,59 % | 2,17 % | 0,11 % | 0,21 % | 0,51 % |
| **25 -** | **26 Years** | 3,87 % | 1,66 % | 2,14 % | 0,12 % | 0,22 % | 0,53 % |
| **26 -** | **27 Years** | 4,27 % | 1,72 % | 2,11 % | 0,12 % | 0,23 % | 0,55 % |
| **27 -** | **28 Years** | 4,61 % | 1,77 % | 2,08 % | 0,13 % | 0,24 % | 0,57 % |
| **28 -** | **29 Years** | 4,90 % | 1,81 % | 2,05 % | 0,14 % | 0,25 % | 0,59 % |
| **29 -** | **30 Years** | 5,13 % | 1,85 % | 2,02 % | 0,14 % | 0,27 % | 0,62 % |
| **30 -** | **31 Years** | 5,32 % | 1,87 % | 1,99 % | 0,15 % | 0,28 % | 0,64 % |
| **31 -** | **32 Years** | 5,46 % | 1,90 % | 1,96 % | 0,16 % | 0,29 % | 0,67 % |
| **32 -** | **33 Years** | 5,57 % | 1,91 % | 1,94 % | 0,17 % | 0,31 % | 0,69 % |
| **33 -** | **34 Years** | 5,64 % | 1,93 % | 1,91 % | 0,17 % | 0,32 % | 0,72 % |
| **34 -** | **35 Years** | 5,68 % | 1,94 % | 1,88 % | 0,18 % | 0,34 % | 0,75 % |
| **35 -** | **36 Years** | 5,70 % | 1,94 % | 1,85 % | 0,19 % | 0,35 % | 0,78 % |
| **36 -** | **37 Years** | 5,70 % | 1,95 % | 1,83 % | 0,20 % | 0,37 % | 0,81 % |
| **37 -** | **38 Years** | 5,68 % | 1,95 % | 1,80 % | 0,21 % | 0,39 % | 0,84 % |
| **38 -** | **39 Years** | 5,65 % | 1,95 % | 1,78 % | 0,22 % | 0,41 % | 0,87 % |
| **39 -** | **40 Years** | 5,61 % | 1,95 % | 1,75 % | 0,23 % | 0,43 % | 0,90 % |
| **40 -** | **41 Years** | 5,55 % | 1,94 % | 1,73 % | 0,24 % | 0,45 % | 0,94 % |
| **41 -** | **42 Years** | 5,49 % | 1,94 % | 1,70 % | 0,26 % | 0,47 % | 0,98 % |
| **42 -** | **43 Years** | 5,42 % | 1,93 % | 1,68 % | 0,27 % | 0,49 % | 1,01 % |
| **43 -** | **44 Years** | 5,35 % | 1,92 % | 1,65 % | 0,28 % | 0,52 % | 1,05 % |
| **44 -** | **45 Years** | 5,27 % | 1,91 % | 1,63 % | 0,30 % | 0,54 % | 1,09 % |
| **45 -** | **46 Years** | 5,19 % | 1,90 % | 1,61 % | 0,31 % | 0,57 % | 1,14 % |
| **46 -** | **47 Years** | 5,11 % | 1,89 % | 1,58 % | 0,33 % | 0,60 % | 1,18 % |
| **47 -** | **48 Years** | 5,03 % | 1,88 % | 1,56 % | 0,34 % | 0,63 % | 1,23 % |
| **48 -** | **49 Years** | 4,95 % | 1,87 % | 1,54 % | 0,36 % | 0,66 % | 1,27 % |
| **49 -** | **50 Years** | 4,87 % | 1,86 % | 1,52 % | 0,38 % | 0,69 % | 1,32 % |
| **50 -** | **51 Years** | 4,78 % | 1,85 % | 1,50 % | 0,40 % | 0,72 % | 1,37 % |
| **51 -** | **52 Years** | 4,70 % | 1,83 % | 1,47 % | 0,42 % | 0,76 % | 1,43 % |
| **52 -** | **53 Years** | 4,62 % | 1,82 % | 1,45 % | 0,44 % | 0,80 % | 1,48 % |
| **53 -** | **54 Years** | 4,54 % | 1,81 % | 1,43 % | 0,46 % | 0,83 % | 1,54 % |
| **54 -** | **55 Years** | 4,47 % | 1,79 % | 1,41 % | 0,48 % | 0,88 % | 1,60 % |
| **55 -** | **56 Years** | 4,39 % | 1,78 % | 1,39 % | 0,51 % | 0,92 % | 1,66 % |
| **56 -** | **57 Years** | 4,31 % | 1,77 % | 1,37 % | 0,53 % | 0,96 % | 1,72 % |
| **57 -** | **58 Years** | 4,24 % | 1,75 % | 1,35 % | 0,56 % | 1,01 % | 1,79 % |
| **58 -** | **59 Years** | 4,17 % | 1,74 % | 1,33 % | 0,58 % | 1,06 % | 1,86 % |
| **59 -** | **60 Years** | 4,10 % | 1,72 % | 1,31 % | 0,61 % | 1,11 % | 1,93 % |
| **60 -** | **61 Years** | 4,03 % | 1,71 % | 1,30 % | 0,64 % | 1,17 % | 2,01 % |
| **61 -** | **62 Years** | 3,96 % | 1,70 % | 1,28 % | 0,68 % | 1,22 % | 2,08 % |
| **62 -** | **63 Years** | 3,90 % | 1,68 % | 1,26 % | 0,71 % | 1,28 % | 2,16 % |
| **63 -** | **64 Years** | 3,83 % | 1,67 % | 1,24 % | 0,74 % | 1,34 % | 2,25 % |
| **64 -** | **65 Years** | 3,77 % | 1,65 % | 1,22 % | 0,78 % | 1,41 % | 2,34 % |
| **65 -** | **66 Years** | 3,71 % | 1,64 % | 1,21 % | 0,82 % | 1,48 % | 2,43 % |
| **66 -** | **67 Years** | 3,65 % | 1,63 % | 1,19 % | 0,86 % | 1,55 % | 2,52 % |
| **67 -** | **68 Years** | 3,59 % | 1,61 % | 1,17 % | 0,90 % | 1,63 % | 2,62 % |
| **68 -** | **69 Years** | 3,54 % | 1,60 % | 1,16 % | 0,95 % | 1,70 % | 2,72 % |
| **69 -** | **70 Years** | 3,48 % | 1,58 % | 1,14 % | 1,00 % | 1,79 % | 2,82 % |
| **70 -** | **71 Years** | 3,43 % | 1,57 % | 1,12 % | 1,04 % | 1,87 % | 2,93 % |
| **71 -** | **72 Years** | 3,38 % | 1,56 % | 1,11 % | 1,10 % | 1,97 % | 3,04 % |
| **72 -** | **73 Years** | 3,33 % | 1,54 % | 1,09 % | 1,15 % | 2,06 % | 3,16 % |
| **73 -** | **74 Years** | 3,28 % | 1,53 % | 1,08 % | 1,21 % | 2,16 % | 3,28 % |
| **74 -** | **75 Years** | 3,23 % | 1,52 % | 1,06 % | 1,27 % | 2,27 % | 3,40 % |
| **75 -** | **76 Years** | 3,18 % | 1,50 % | 1,04 % | 1,33 % | 2,38 % | 3,53 % |
| **76 -** | **77 Years** | 3,14 % | 1,49 % | 1,03 % | 1,39 % | 2,49 % | 3,67 % |
| **77 -** | **78 Years** | 3,09 % | 1,48 % | 1,02 % | 1,46 % | 2,61 % | 3,81 % |
| **78 -** | **79 Years** | 3,05 % | 1,47 % | 1,00 % | 1,54 % | 2,74 % | 3,96 % |
| **79 -** | **80 Years** | 3,01 % | 1,45 % | 0,99 % | 1,61 % | 2,87 % | 4,11 % |
| **80 -** | **81 Years** | 2,97 % | 1,44 % | 0,97 % | 1,69 % | 3,01 % | 4,26 % |
| **81 -** | **82 Years** | 2,93 % | 1,43 % | 0,96 % | 1,77 % | 3,15 % | 4,43 % |
| **82 -** | **83 Years** | 2,89 % | 1,42 % | 0,94 % | 1,86 % | 3,31 % | 4,60 % |
| **83 -** | **84 Years** | 2,85 % | 1,41 % | 0,93 % | 1,95 % | 3,47 % | 4,77 % |
| **84 -** | **85 Years** | 2,81 % | 1,39 % | 0,92 % | 2,05 % | 3,63 % | 4,95 % |
| **85 -** | **86 Years** | 2,77 % | 1,38 % | 0,90 % | 2,15 % | 3,81 % | 5,14 % |
| **86 -** | **87 Years** | 2,74 % | 1,37 % | 0,89 % | 2,26 % | 3,99 % | 5,33 % |
| **87 -** | **88 Years** | 2,70 % | 1,36 % | 0,88 % | 2,37 % | 4,18 % | 5,54 % |
| **88 -** | **89 Years** | 2,67 % | 1,35 % | 0,87 % | 2,48 % | 4,38 % | 5,75 % |
| **89 -** | **90 Years** | 2,64 % | 1,34 % | 0,85 % | 2,60 % | 4,59 % | 5,96 % |
| **90 -** | **91 Years** | 2,60 % | 1,33 % | 0,84 % | 2,73 % | 4,81 % | 6,19 % |
| **91 -** | **92 Years** | 2,57 % | 1,32 % | 0,83 % | 2,87 % | 5,04 % | 6,42 % |
| **92 -** | **93 Years** | 2,54 % | 1,31 % | 0,82 % | 3,01 % | 5,28 % | 6,66 % |
| **93 -** | **94 Years** | 2,51 % | 1,29 % | 0,81 % | 3,15 % | 5,53 % | 6,91 % |
| **94 -** | **95 Years** | 2,48 % | 1,28 % | 0,80 % | 3,31 % | 5,80 % | 7,17 % |
| **95 -** | **96 Years** | 2,45 % | 1,27 % | 0,78 % | 3,47 % | 6,07 % | 7,44 % |
| **96 -** | **97 Years** | 2,42 % | 1,26 % | 0,77 % | 3,64 % | 6,36 % | 7,72 % |
| **97 -** | **98 Years** | 2,40 % | 1,25 % | 0,76 % | 3,81 % | 6,66 % | 8,01 % |
| **98 -** | **99 Years** | 2,37 % | 1,24 % | 0,75 % | 4,00 % | 6,98 % | 8,31 % |
| **99 -** | **100 Years** | 2,34 % | 1,23 % | 0,74 % | 4,20 % | 7,31 % | 8,62 % |

# Supplementary Appendix 6 - validation of the model.

## Face validity

We have had a close cooperation between modelers and clinicians through all steps of the modeling, from conception, statistical analyses and estimation of input parameters, and valuation of the final model. This has enabled clinicians to give input regarding whether the model aligns with their knowledge from the clinic. Clinicians confirm the models face validity regarding the high transition during childhood years, and regarding the high healthcare costs at older ages based on their knowledge regarding disease risk of obesity.

## Internal validity

Internal validation, where we examined the mathematical calculations used in the model, was performed in several ways:

1. We used several control cells in the models work sheet. For example, we ensured that the cohort in the model was constant in the different cycles.
2. We ensured that the equations we used to estimate transition probabilities in the model were correct by ensuring that the predicted time to event from the statistical model (in STATA) was equal to the predicted time-to-event (based on the equations used in Excel).
3. We ran a number of tests where input parameters were set at 1 or 0, to see that the results from the model would change according to our expectations. As an example, when we set all transition probabilities between Obese grade 1 and Obese grade 2 to zero (scenario 1, when estimating expected survival if eliminating obesity grade 2) the cost of obesity grade 2 reduced to zero, as expected.
4. The first author (Bjørnelv) built the model, while the senior author (Halsteinli) did a thorough evaluation of the mathematical estimates in the model.

In addition, we did a walk-through of the model in September 2019 with experienced modelers in another field (industrial economics). The aim was to search for errors in the logic and mathematical calculations in the model. The external modelers confirmed the models’ internal validity.

## External validity, dependent comparison 1

**Comparison between the MOON-model and input-data (children)**

To test the internal validity of the childhood-part of the model, we ran survival analyses using the data from the Child-growth study (CGS) alone to estimate transition probabilities. We then used these transition probabilities as input in the model, and compared the output of the model with prevalence estimated from the CGS. Results can be seen in Table S18 and S19.

When using data from the CGS alone, the output from the model gives very similar prevalence estimates as the data used to inform the model. This confirms the validity of the model, and the statistical methodology used to estimate transition probabilities (see Statistical analyses in the main text of the article).

Table S18: estimates of the prevalence of normal weight, overweight, obesity grade 1 and obesity grade 2 in the child-growth study.

| **CGS** | **Normal weight** | **Overweight** | **Obese grade 1** | **Obese grade 2** |
| --- | --- | --- | --- | --- |
| **Age cohort (all)** |  |  |  |  |
| **2 years** | 0.90 | 0.09 | 0.01 | 0.00 |
| **3 years** | 0.90 | 0.09 | 0.01 | 0.00 |
| **4 years** | 0.89 | 0.09 | 0.01 | 0.00 |
| **5 years** | 0.87 | 0.11 | 0.01 | 0.00 |
| **6 years** | 0.84 | 0.14 | 0.02 | 0.00 |
| **7 years** | 0.81 | 0.16 | 0.02 | 0.00 |
| **8 years** | 0.78 | 0.19 | 0.03 | 0.00 |

Table s19: estimates of the prevalence of normal weight, overweight, obesity grade 1 and obesity grade 2 in the MOON-study, when only using data from the child-growth study as input in the model.

| **MOON output** | **Normal weight** | **Overweight** | **Obese grade 1** | **Obese grade 2** |
| --- | --- | --- | --- | --- |
| **Age cohort (all)** |  |  |  |  |
| 2 years | 0.90 | 0.09 | 0.01 | 0.00 |
| 3 years | 0.89 | 0.08 | 0.02 | 0.01 |
| 4 years | 0.90 | 0.09 | 0.01 | 0.00 |
| 5 years | 0.88 | 0.10 | 0.02 | 0.01 |
| 6 years | 0.85 | 0.10 | 0.03 | 0.01 |
| 7 years | 0.83 | 0.13 | 0.03 | 0.01 |
| 8 years | 0.79 | 0.17 | 0.04 | 0.01 |

## External validity, dependent comparison 2

**Comparison between the MOON and the input-data (adults)**

Comparison to HUNT 3 (see Midthjelle et al. 2013) (8).

In testing the external validity of the adulthood-part of the MOON study, we compared its estimated prevalence with that of HUNT 3 (the most recent health survey among the surveys we used as input in the model). Since our model is built using data from the CGS, Trondheim municipality, and HUNT 1, 2 and 3, the output is not expected to be the same as in HUNT 3 (as expected in the childhood validation). However, we are testing to see whether the curves look similar (prevalence at different ages), and whether the prevalence numbers are reasonable considering the input in the model.

First, we checked the prevalence of normal weight, overweight, obese grade 1 and obese grade 2 in the entire cohort in the HUNT 3 and for all ages in the MOON (see Table S20). The prevalence predicted corresponds well to the prevalence observed in the HUNT 3.

**Table s20: estimates of the prevalence of normal weight, overweight, obesity grade 1 and obesity grade 2 in the MOON-study compared to that observed for the whole population in HUNT 3.**

| **HUNT 3** | | | | | **MOON** | | | | |
| --- | --- | --- | --- | --- | --- | --- | --- | --- | --- |
| **Age groups** | **NW** | **OW** | **OB1** | **OB2** | **Age groups** | **NW** | **OW** | **OB 1** | **OB 2** |
| Men (20 +) | 0.26 | 0.52 | 0.19 | 0.03 | Men (20 +) | 0.30 (0.27-0.32) | 0.47 (0.45-0.49) | 0.17 (0.16-0.19) | 0.05 (0.04 – 0.07) |
| Women (20 +) | 0.40 | 0.38 | 0.17 | 0.05 | Women (20 +) | 0.36 (0.33-0.38) | 0.35 (0.33-0.37) | 0.21 (0.20-0.23) | 0.08 (0.06-0.09) |

Second, we checked the prevalence of normal weight, overweight, obesity (BMI ≥ 30) at different ages for males and females in the HUNT 3, and for males and females estimated from the MOON. Results can be seen in Table S21 and Figures S2 and S3.

**Table s21: estimates of the prevalence of normal weight, overweight, obesity in the MOON-study compared to the HUNT 3 study.**

| **HUNT 3** | | | | **MOON** | | | |
| --- | --- | --- | --- | --- | --- | --- | --- |
| **Age groups** | **NW** | **OW** | **OB** | **Age groups** | **NW** | **OW** | **OB** |
| Men (20-29) | 0.52 | 0.35 | 0.13 | Men (20-29) | 0.71 (0.65-0.75) | 0.24 (0.20-0.29) | 0.05 (0.04-0.08) |
| Men (30-39) | 0.27 | 0.52 | 0.21 | Men (30-39) | 0.44 (0.40-0.47) | 0.44 (0.42-0.47) | 0.11 (0.09-0.14) |
| Men (40-49) | 0.22 | 0.54 | 0.24 | Men (40-49) | 0.26 (0.24-0.28) | 0.54 (0.52-0.55) | 0.20 (0.18-0.23) |
| Men (50-59) | 0.21 | 0.55 | 0.24 | Men (50-59) | 0.18 (0.16-0.19) | 0.54 (0.52-0.55) | 0.29 (0.27-0.31) |
| Men (60-69) | 0.20 | 0.55 | 0.25 | Men (60-69) | 0.15 (0.14-0.16) | 0.51 (0.50-0.53) | 0.34 (0.32-0.36) |
| Men (70-79) | 0.26 | 0.53 | 0.21 | Men (70-79) | 0.15 (0.14-0.16) | 0.49 (0.48-0.51) | 0.36 (0.33-0.38) |
| Men (80+) | 0.36 | 0.49 | 0.15 | Men (80+) | 0.26 (0.22-0.29) | 0.50 (0.47-0.53) | 0.25 (0.20-0.30) |
|  |  |  |  |  |  |  |  |
| Women (20-29) | 0.62 | 0.25 | 0.13 | Women (20-29) | 0.75 (0.71-0.79) | 0.19 (0.16-0.23) | 0.05 (0.04-0.08) |
| Women (30-39) | 0.49 | 0.31 | 0.20 | Women (30-39) | 0.59 (0.56-0.62) | 0.30(0.27-0.32) | 0.11 (0.09-0.14) |
| Women (40-49) | 0.42 | 0.37 | 0.21 | Women (40-49) | 0.41 (0.38-0.43) | 0.38 (0.37-0.40) | 0.21 (0.19-0.23) |
| Women (50-59) | 0.36 | 0.41 | 0.23 | Women (50-59) | 0.28 (0.26-0.29) | 0.41 (0.40-0.42) | 0.31 (0.29-0.34) |
| Women (60-69) | 0.30 | 0.42 | 0.28 | Women (60-69) | 0.21 (0.20-0.23) | 0.40 (0.38-0.41) | 0.39 (0.37-0.41) |
| Women (70-79) | 0.28 | 0.42 | 0.30 | Women (70-79) | 0.19 (0.17-0.20) | 0.38 (0.36-0.40) | 0.44 (0.41-0.46) |
| Women (80+) | 0.32 | 0.44 | 0.24 | Women (80+) | 0.23 (0.20-0.25) | 0.38 (0.36-0.40) | 0.39 (0.35-0.44) |

Interpretation: The curves of the prevalence of normal weight, overweight and obesity show similar patterns when estimated from the MOON and HUNT, Figures 6 (main paper). The prevalence of overweight was similar between the HUNT 3 and the output from the MOON, for both males and females and in all age groups. However, in younger age groups, the MOON predicts higher rates of normal weight and lower rates of obesity, compared to the HUNT-3. This pattern changes as the cohort ages, where the MOON predicts lower rates of normal weight and higher rates of obesity compared to that observed in the HUNT 3.

The prevalence of overweight increased between HUNT 1 and HUNT2, but levelled off between HUNT 2 and HUNT 3: this explains why the model predicts equal rates of overweight as that observed in HUNT 3. However, the rate of obesity increased significantly both between HUNT 1 and HUNT2, and between HUNT 2 and HUNT 3: for example, for men in the age groups 60-69 and 70-79, the prevalence of obesity increased by 6 and 5 percent points between HUNT 1 and HUNT 2, and continued to increase by 7 percent points and 5 percent points between HUNT 2 and HUNT 3, respectively (8). Consequently, the MOON is predicting the prevalence of overweight and obesity in the future *given* that the development in overweight and obesity continues as it has been observed to do between 1980s (HUNT 1) and the 2000s (HUNT 3) for all age groups.

References

1. [Global BMI Mortality Collaboration](https://www.ncbi.nlm.nih.gov/pubmed/?term=Global%20BMI%20Mortality%20Collaboration%5BAuthor%5D&cauthor=true&cauthor_uid=27423262), [Di Angelantonio E](https://www.ncbi.nlm.nih.gov/pubmed/?term=Di%20Angelantonio%20E%5BAuthor%5D&cauthor=true&cauthor_uid=27423262), [Bhupathiraju ShN](https://www.ncbi.nlm.nih.gov/pubmed/?term=Bhupathiraju%20ShN%5BAuthor%5D&cauthor=true&cauthor_uid=27423262), [Wormser D](https://www.ncbi.nlm.nih.gov/pubmed/?term=Wormser%20D%5BAuthor%5D&cauthor=true&cauthor_uid=27423262), [Gao P](https://www.ncbi.nlm.nih.gov/pubmed/?term=Gao%20P%5BAuthor%5D&cauthor=true&cauthor_uid=27423262), [Kaptoge S](https://www.ncbi.nlm.nih.gov/pubmed/?term=Kaptoge%20S%5BAuthor%5D&cauthor=true&cauthor_uid=27423262). Body-mass index and all-cause mortality: individual-participant-data meta-analysis of 239 prospective studies in four continents. [Lancet.](https://www.ncbi.nlm.nih.gov/pubmed/27423262) 2016 Aug 20;388(10046):776-86. doi: 10.1016/S0140-6736(16)30175-1. Epub 2016 Jul 13.
2. Lindahl AK. International Profiles of Health Care Systems. The Norwegian health care system. The Commonwealth Fund. 2017. Available from: <http://www.commonwealthfund.org/publications/fund-reports/2017/may/international-profiles>
3. The Norwegian Directorate of Health. Innsatsstyrt finansiering 2009 [Activity based funding 2009]. (2009). Available from: <https://www.helsedirektoratet.no/tema/finansiering>.
4. Bremnes R, Halsteinli V, Rønningsen S., Kalseth J and Bjørngaard JH. Aktivitetsmål og enhetskostnader for psykisk helsevern i Helse Midt-Norge RHF. SINTEF Rapport. ISSN: 1504-9795. ISBN: 9788214046670. Available from: <https://www.sintef.no/publikasjoner/publikasjon/?pubid=CRIStin+1269487>
5. The Norwegian Directorate of Health. SAMDATA Spesialisthelsetjenesten. 2003-2017. Nr. 10/2018. Available from: <https://www.helsedirektoratet.no/statistikk/statistikk/samdata-spesialisthelsetjenesten/analysenotater-samdata-spesialisthelsetjenesten/2018-10%20Hovedresultater%20Samdata%20spesialisthelsetjenesten%202013-2017.pdf/_/attachment/inline/d664518e-04e6-4d08-ab22-37c6928beb56:ecab416df070be8b4870be085bbcaf3dddc315f3/2018-10%20Hovedresultater%20Samdata%20spesialisthelsetjenesten%202013-2017.pdf>
6. The Norwegian Directorate of Health. KUHR-databasen [The KUHR database]. (2017). Available from: <https://helsedirektoratet.no/kuhr-databasen>
7. The Norwegian Directorate of Health. Økonomisk evaluering av helsetiltak – en veileder. IS-1985. Available from: <https://www.helsedirektoratet.no/veiledere/okonomisk-evaluering-av-helsetiltak/%C3%98konomisk%20evaluering%20av%20helsetiltak%20%E2%80%93%20Veileder.pdf/_/attachment/inline/a4c0d8f4-6420-47a5-a052-344a5f2d0e76:637c45a5f29eda29afb9625968759e6fa4c4af61/%C3%98konomisk%20evaluering%20av%20helsetiltak%20%E2%80%93%20Veileder.pdf>.
8. K Midthjell, C M Y Lee, A Langhammer, S Krokstad, T L Holmen, K Hveem, S Colagiuri, and J Holmen. Trends in overweight and obesity over 22 years in a large adult population: the HUNT Study, Norway. Clin Obes. 2013 Feb; 3(1-2): 12–20. Published online 2013 Mar 19.
