# Supplementary material for: Modeling Obesity in Norway (The MOON Study): A Decision-Analytic Approach—Prevalence, Costs, and Years of Life Lost
Source: Med Decis Making. 2020 Nov 30;41(1):21–36. doi: 10.1177/0272989X20971589 (PMC7783689; doi:10.1177/0272989X20971589)
Supplement: sj-docx-1-mdm-10.1177_0272989X20971589 – Supplemental material for Modeling Obesity in Norway (The MOON Study): A Decision-Analytic Approach—Prevalence, Costs, and Years of Life Lost [file sj-docx-1-mdm-10.1177_0272989X20971589.docx]

October 6^th^ 2020

Trondheim

Dear Editor,

Thank you once again for the news regarding the acceptance of our submitted article to Medical Decision Making, titled *“Modeling Obesity in Norway (The MOON Study): A Decision-Analytic Approach - Prevalence, Costs, and Years-of-Life Lost".*

We thank the reviewers for valuable comments and inputs for improvements of the paper. We have tried to respond accordingly.

We would like our manuscript to have color images in the print version, and also to be open access. We accept the additional costs for these features – and an invoice can be sent to:

Obece v/Bård Kulseng

St. Olavs hospital HF
Fakturamottak
Postboks 2622
6405 Molde

Sincerely yours,

Gudrun Maria Waaler Bjørnelv

(Corresponding author)
